# Mapping the Binding Interactions between Human Gasdermin D and Human Caspase-1 Using Carbene Footprinting

**DOI:** 10.1021/jacsau.3c00236

**Published:** 2023-06-23

**Authors:** James R. Lloyd, Antonio Biasutto, Katharina L. Dürr, Ali Jazayeri, Jonathan T.S. Hopper, Neil J. Oldham

**Affiliations:** † School of Chemistry, University of Nottingham, University Park, Nottingham NG7 2RD, U.K.; ‡ OMass Therapeutics, Schrodinger Building, Oxford Science Park, Oxford OX4 4GE, U.K.

**Keywords:** carbene labeling, diazirines, protein footprinting, gasdermin D, caspase-1, pyroptosis

## Abstract

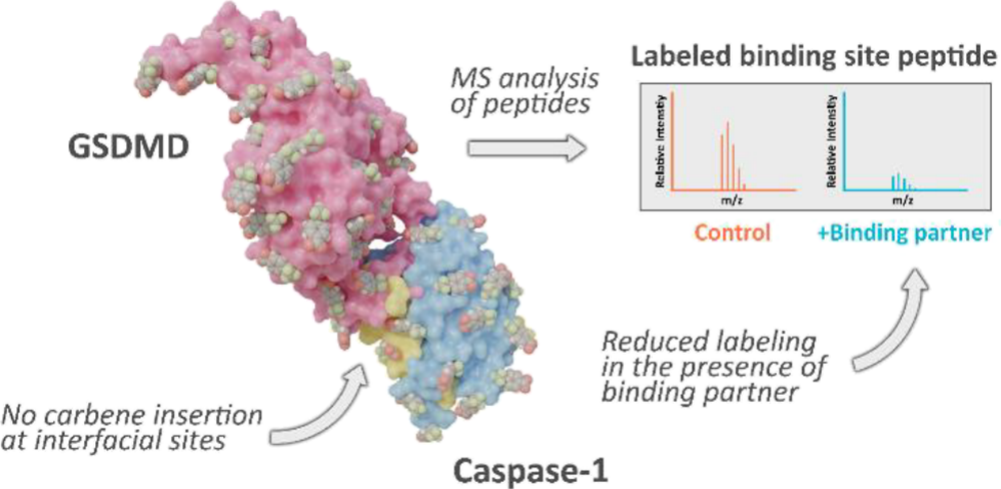

Carbene footprinting is a recently developed mass spectrometry-based chemical labeling technique that probes protein interactions and conformation. Here, we use the methodology to investigate binding interactions between the protease human Caspase-1 (C285A) and full-length human Gasdermin D (hGSDMD), which are important in inflammatory cell death. GSDMD is cleaved by Caspase-1, releasing its N-terminal domain which oligomerizes in the membrane to form large pores, resulting in lytic cell death. Regions of reduced carbene labeling (masking), caused by protein binding, were observed for each partner in the presence of the other and were consistent with hCaspase-1 exosite and active-site interactions. Most notably, the results showed direct occupancy of hCaspase-1 (C285A) active-site by hGSDMD for the first time. Differential carbene labeling of full-length hGSDMD and the pore-forming N-terminal domain assembled in liposomes showed masking of the latter, consistent with oligomeric assembly and insertion into the lipid bilayer. Interactions between Caspase-1 and the specific inhibitor VRT-043198 were also studied by this approach. In wild-type hCaspase-1, VRT-043198 modifies the active-site Cys285 through the formation of a S,O-hemiacetal. Here, we showed by carbene labeling that this inhibitor can noncovalently occupy the active site of a C285A mutant. These findings add considerably to our knowledge of the hCaspase-1-hGSDMD system.

## 1 Introduction

Methods that allow the detection and mapping of interactions between proteins, and between proteins and their ligands, are vital both for understanding biology at the molecular level and for drug discovery efforts. High-resolution techniques, such as X-ray crystallography and nuclear magnetic resonance (NMR) spectroscopy, are widely used for these purposes. They provide atomic-level details but suffer from relatively low sensitivity and slow turn-around times. Structural mass spectrometry-based approaches are increasingly used to overcome these two hurdles by exploiting the analytical speed and sensitivity afforded by mass spectrometry (MS). Native electrospray (ESI)-MS enables the direct detection of protein-ligand complexes and hence a method for screening drug binding to a target protein.^1^ The ability to map ligand binding sites is also offered by MS, providing a suitable reporter for the binding-induced change in solvent accessibility of a protein surface is used.^2^ The most established method for this is hydrogen-deuterium exchange (HDX).^3^ The mass shift caused by the exchange of backbone amide N–H protons with deuterium from ^2^H_2_O is readily detected by MS and can be mapped to the peptide level by use of appropriate proteases and liquid chromatography (LC)-ESI-MS conditions. HDX is thus able to detect masking/unmasking induced by ligand binding, protein conformational changes, and protein folding/unfolding processes.

In addition to HDX, covalent protein labeling methods are also able to report masking and unmasking events on a protein surface. The relatively slow rate of standard covalent labeling can be problematic for more dynamic systems and so effort has been directed to “fast” labeling using photochemically activated probes. Fast photochemical oxidation of proteins (FPOP) is one such method, which uses OH radicals to label proteins.^4^ Produced by the homolytic cleavage of H_2_O_2_ following irradiation of the sample with UV radiation at ca. 266 nm, OH radicals oxidize accessible amino acid side chains to induce +14, +16, and +32 Da mass shifts. Any changes in accessibility caused by binding or folding events may then be inferred from changes in the extent of oxidation in particular regions of the protein.

**Scheme 1:**
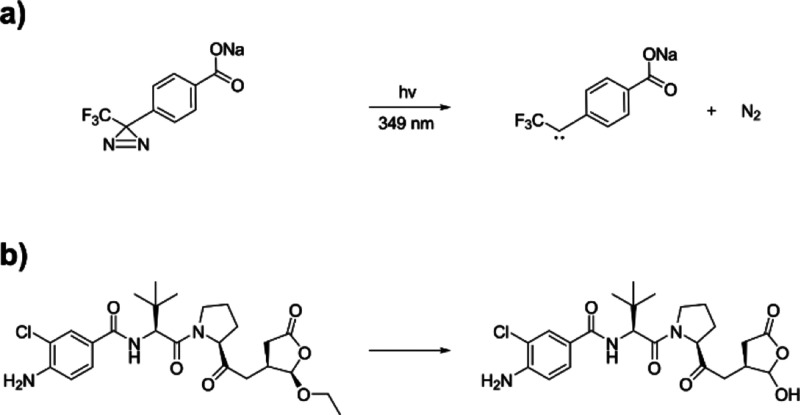
(a) Photoactivation of NaTDB to the Carbene Species and (b) Conversion of Belnacasan (VX-765) to VRT-043198

An interesting alternative to FPOP is carbene labeling of proteins.^5^ Carbenes are most conveniently generated by photolysis of an organic diazirene at ca. 350 nm. Attachment of the carbene, either by addition or insertion mechanisms, results in a clear and substantial protein mass shift, corresponding to the mass of the carbene generated, which is typically 100–200 Da per label. Carbenes are frequently employed in photoaffinity cross-linking experiments (alongside aromatic ketones, azides, and diazoalkanes) to covalently anchor ligands to their biological targets but their application as footprinting probes is becoming increasingly popular.^6^ The irreversible nature of covalent labeling means that these approaches enjoy some advantages over HDX. First, the labeled protein requires no special treatment to prevent back-exchange of the label and can be separated from a complex mixture using sodium dodecyl sulfate-polyacrylamide gel electrophoresis (SDS-PAGE), if required.^7^ Second, a full range of proteases can be employed to digest the sample (not just those that function at the low pH quenching conditions required for HDX).^8^ Third, LC-ESI-MS analysis can be run at normal temperatures, and fourth, collision-induced dissociation (CID) MS/MS fragmentation of peptide ions can be performed without the deuterium scrambling issues seen with HDX. This allows subpeptide analysis of labeling to identify masking or unmasking at resolution approaching the amino acid level.^8^ Our diazirene probe of choice is sodium 4-[3-(trifluoromethyl)-3H-diazirin-3-yl]benzoate (NaTDB).^9^ This efficiently labels a wide range of proteins following activation at 350 nm and resulting in a 202 Da increase in mass (Scheme 1a). Recently, we have successfully employed this diazirene-based carbene probe to map interactions of a range of protein systems.^9−11^

**Figure 1:**
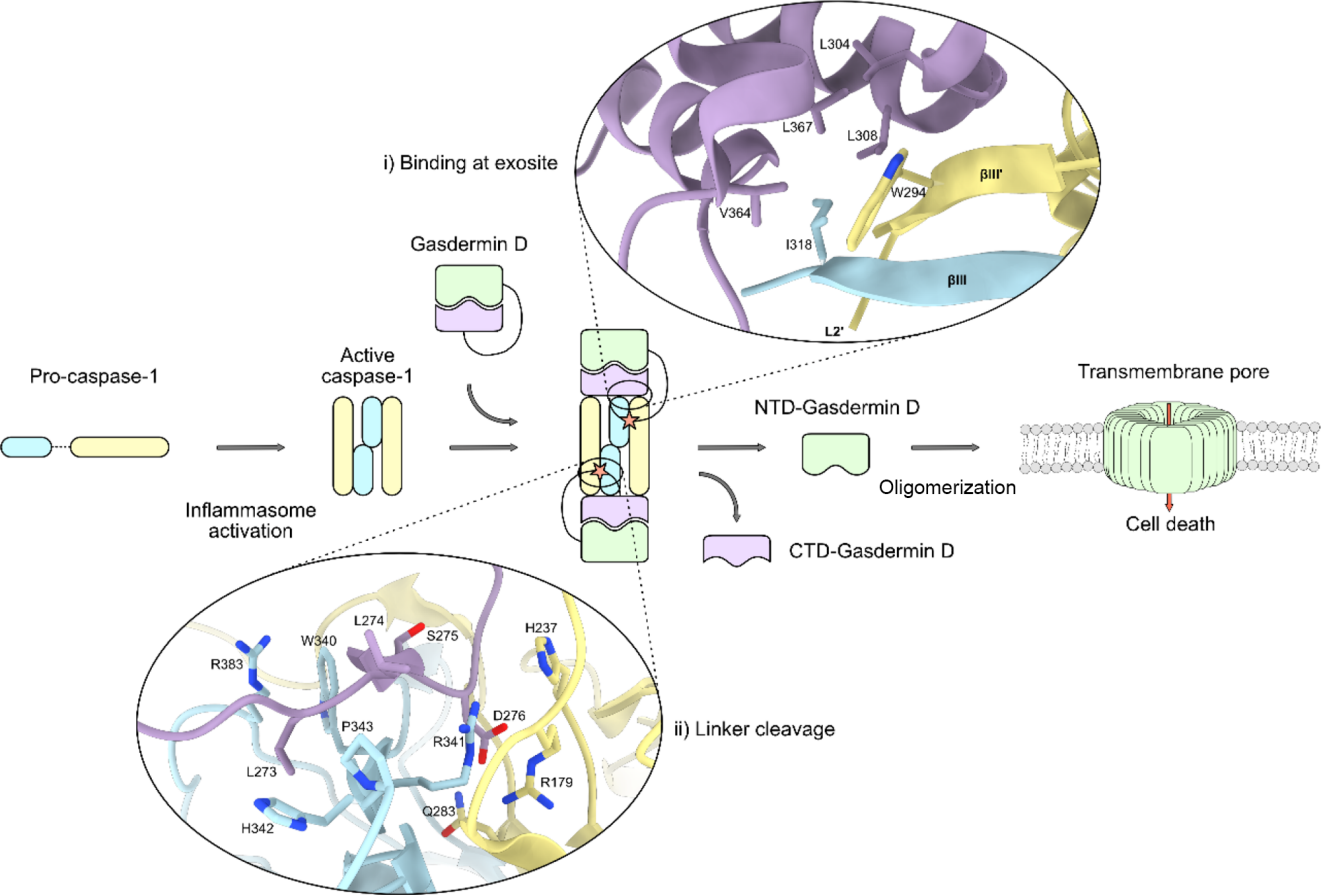
Activation of NTD-Gasdermin D by inflammatory Caspase-1 and subsequent oligomerization and transmembrane-pore formation in the lipid bilayer (human GSDMD/human Caspase-1 exosite-mediated binding adapted from PDB 6KN0, mouse GSDMD/human Caspase-1 linker binding to catalytic domain adapted from PDB 6VIE). GSDMD colored purple, Caspase-1 p20 subunit colored yellow, and Caspase-1 p10 subunit colored light blue. Residues participating in these interfaces are shown.

Gasdermin D (GSDMD) is a pore-forming protein and key initiator of pyroptosis: an inflammatory form of lytic cell death that occurs in response to diverse pathogenic and sterile insults.^12−14^ GSDMD is a 484-residue (53 kDa) protein, encoded for by the *GSDMD* gene and a member of the gasdermin family of proteins. GSDMD is formed of two conserved domains, an N-terminal domain with an extended β-sheet core structure and a C-terminal domain that is further characterized by a linker region (linking N and C termini), a helix repeat-I bundle, a helix repeat-II bundle, and an intermediated β-strand insertion.^15−17^ In the resting state, the C-terminal domain interacts with the N-terminal domain and stabilizes it, autoinhibiting the protein’s pore-forming activity. However, upon appropriate host defense signals formation of the inflammasome leads to activation of inflammatory Caspases (namely, Caspase-1) that cleave GSDMD’s flexible linker. The N-terminal domain of GSDMD oligomerizes at the plasma membrane to form a 33-subunit transmembrane pore, approximately 320 Å in diameter, rupturing the membrane and inducing cell death (Figure 1).^18,19^

Caspase-1 is a cysteine protease formed from a heterodimer of p10 and p20 subunits.^20^ The active enzyme is a dimer of heterodimers where each of the two catalytic domains span p10/p20 interfaces. These are composed of the triad His237, Gly238, and nucleophilic Cys285. Caspase-1 recognizes the tetrapeptide motif XXXD and induces cleavage after the aspartate residue.^21^ The mechanism behind Caspase substrate specificity is poorly understood, although it is recognized that Caspase-1 prefers hydrophobic/aromatic residues at position P4 (according to the Schecter–Berger nomenclature, which describes the enzyme and substrate sites for proteases^22^) and small aliphatic residues at position P1′.^23^ For mGSDMD, this region includes 272-SLLSDGIDE-280, where P4 is Leu273 and P1′ is Gly277.^23^ Whilst the XXXD motif defines a portion of hGSDMD that must interact with Caspase-1, it does not reveal details of other regions of the protein important for enzyme–substrate interactions.

Pyroptosis is recognized as a contributor to many human diseases, including cancer and inflammatory disorders. Inhibition of GSDMD activation is therefore an attractive therapeutic strategy.^24^ Belnacasan (VX-765) is a pro-drug and Caspase inhibitor,^25^ which forms the active drug VRT-043198 (O-desethyl-belnacasan) upon esterase cleavage of VX-765 (Scheme 1b). A potent electrophile, VRT-043198, can modify the catalytic Cys285 thiol, thus impeding Caspase activity and preventing GSDMD activation. Administration of VX-765 to mice showed decreased lipopolysaccharide-induced cytokine secretion and reduced inflammatory disease severity.^25^ Phase IIa clinical trials of VX-765 were discontinued due to liver toxicity; however, development of structurally similar Caspase-targeting drugs continues.^26^

Structural elucidation of the full-length human GSDMD-human Caspase-1 complex (hGSDMD/hCaspase-1) has proved difficult owing to the structural heterogeneity and flexibility of GSDMD. Recently, Wang and colleagues determined a structure of the complex between the C-terminal domain (CTD) of hGSDMD and hCaspase-1.^27^ This showed 2:2 binding stoichiometry of GSDMD–Caspase-1. It also highlighted the importance of the Caspase-1 βIII/βIII′ sheet in mediating complex formation through its insertion into a hydrophobic groove on the CTD-GSDMD. Leu304, Leu308, Val364, and Val367 on hGSDMD were shown to make hydrophobic contacts with Trp294 and Ile318 on Caspase-1, and importantly, GSDMD-Caspase-1 binding was shown to be independent of the tetrapeptide cleavage site.^27^ Unfortunately, the flexible linker bearing this motif was not seen in the structure. Liu and colleagues showed that mouse GSDMD/human Caspase-1 (mGSDMD/hCaspase-1) interaction was mediated through the same hydrophobic exosite contacts.^23^ This structure did include a truncated form of mGSDMD’s linker loop and revealed Asp276, of the tetrapeptide, LLSD, buried into the Caspase active site. The authors also reported the P4 site residue _Gas_Leu273 contacting _Casp_Arg383, Trp340, and His342. His342 was also hydrogen-bonded to the P5 site residue _Gas_Ser272. At the P1′ site, a main-chain hydrogen bond between _Gas_Gly277 and _Casp_His237 further anchored the linker to the catalytic groove whilst providing the conformational flexibility for the loop to exit the domain.^23^ Despite these two structures providing complementary information into the molecular mechanisms behind GSDMD binding and activation, mouse and human GSDMD constructs only share ∼60% sequence identity. As such, it is not known whether hGSDMD/hCaspase-1 make the same linker contacts to the catalytic domain of Caspase-1. Therefore, given the difficulty in generating crystal structures of full-length hGSDMD, we sought to further characterize the binding interactions between it and hCaspase-1.

Here, we report the use of carbene footprinting to study three key aspects of the hGSDMD system. First, we provide an accurate map of the interactions between full-length hGSDMD and hCaspase-1 (C285A), including those at the active site as well as exosite of hCaspase-1 (C285A). Second, we show changes in carbene labeling within the N-terminus of hGSDMD upon cleavage by hCaspase-1 (C285A) and associated pore formation in liposomes. Third, we detect and map noncovalent binding of the Caspase-1 inhibitor VRT-043198 to the active site of hCaspase-1 (C285A), providing evidence for the potential of these compounds as noncovalent inhibitors of hCaspase-1.

## 2 Results and Discussion

### 2.1 Optimization of Sequence Coverage and Labeling

Following successful expression of full-length hGSDMD and hCaspase-1 (C285A), we sought to optimize GSDMD and Caspase-1 (C285A) digestion and carbene labeling conditions. The catalytically inactive mutant was employed throughout this study to prevent enzymatic turnover of GSDMD. Sequence coverage optimization was performed to maximize the number of detectable peptides by LC–MS. Optimization of labeling conditions ensured that peptide-level modification was at an appropriate level to report on differential binding partner masking effects.

In-silico digestion was performed on the sequences of both proteins, and the results are shown in Figures S1–S3. From these findings, trypsin and Glu-C were predicted to produce the best coverage for GSDMD, whilst trypsin, Glu-C, and chymotrypsin generated the most useful peptides for Caspase-1. Tryptic digestion of GSDMD generated 26 peptides corresponding to 61% sequence coverage, with much of the C-terminus remaining unrepresented (Figure S4). This region was recognized to contain a high Glu content and showed good theoretical coverage by Glu-C in in silico digestion. When digested with Glu-C, GSDMD revealed 29 peptides and a further 25% gain in sequence coverage over the C-terminus (Figure S4). This multi-protease approach was highly beneficial to maximizing sequence coverage but, as with many techniques employed in protein structural study, it was not possible to achieve complete mapping of the protein sequence, which did place a limit on the information obtained. Tryptic digestion of the Caspase-1 p20 subunit generated 8 peptides (Figure S5) corresponding to 79% sequence coverage whilst proteolysis of the p10 subunit gave 80% peptide coverage (Figure S6). Digestion of Caspase-1 with Glu-C and chymotrypsin yielded no improvement in sequence coverage and so trypsin digestion only was employed for Caspase-1.

Carbene labeling (using NaTDB) is typically carried out at low millimolar concentrations (10–50 mM) with most soluble proteins requiring 10–20 mM of the diazirine. Both GSDMD and Caspase-1 showed satisfactory levels of carbene modification at 20 mM NaTDB (Figures S7–S10). Caspase-1 displayed a distinctive labeling footprint (Figure S10). Regions of no labeling were largely mapped to the dimer interface (Figure S11), which may suggest a dimer. Previous work has shown that isolated Caspase-1 is essentially monomeric at low concentration,^28^ and so it would be surprising if the dimer was present in the absence of GSDMD. Native gel electrophoresis of Caspase-1 gave a single band of low mobility (Figure S12), but this may be due to the relatively high pI of hCaspase-1, rather than a multimeric form.

### 2.2 Differential Footprinting of hGSDMD/hCaspase-1

**Figure 2:**
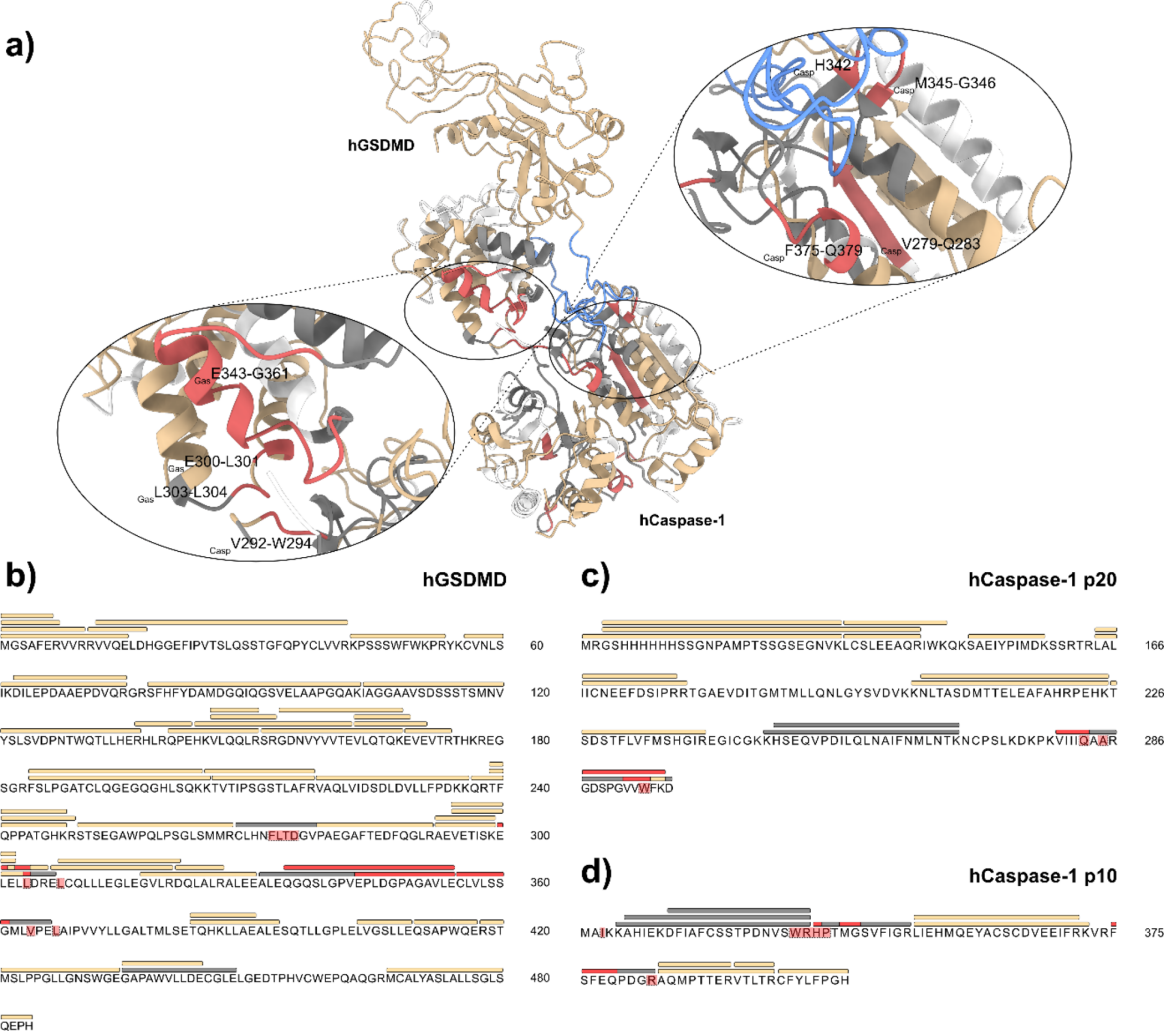
Carbene footprinting of hGSDMD and hCaspase-1. (a) Labeling results mapped onto the full-length hGSDMD-hCaspase-1 complex. Color scheme is as follows: red = masking effect, tan = no change, gray = no labeling, white = no peptide coverage. (b) Labeling results mapped onto the hGSDMD sequence. Bars above the sequence represent peptides, whilst highlighted residues indicate predicted interaction regions. Since MS/MS was performed on selected labeled peptides, higher resolution labeling information is displayed on peptides where available. Coloring is the same as above. (c) Labeling results mapped onto the hCaspase-1 p20 sequence. (d) Labeling results mapped onto the hCaspase-1 p10 sequence.

Following carbene footprinting of GSDMD and Caspase-1 in isolation, labeling was carried out in the presence and absence of 2-fold excess binding partner, as described in the Materials and Methods section. Native gel electrophoresis revealed that a 2-fold excess was required to ensure full complex formation in each case (Figure S12). Additionally, the presence of 20 mM diazirine showed no disruption to the complex, consistent with previous findings by us that NaTDB does not perturb protein–protein or protein-ligand interactions.^9^ Differentially footprinted GSDMD (±Caspase-1, 2-fold excess) revealed a labeling reduction on tryptic peptide 300–306 only in the presence of Caspase-1 (Figures 2 and S13–S16). A similar comparison of Glu-C-derived GSDMD peptides showed that four exhibited a significant decrease in labeling due to Caspase-1 masking, namely, peptides 301–307, 332–354, 335–354, and 355–366 (Figures 2, S17A, and S18). The fact that peptides 300–306 and 301–307, derived from trypsin and Glu-C digestion, respectively, both showed significant reductions in labeling in the presence of Caspase-1 provides mutually supportive data for masking of this region of GSDMD due to Caspase-1 binding (Figure 2). Moreover, labeling reduction in contiguous Glu-C peptides 332–354 and 355–366 shows that the binding interaction also extends over these regions of GSDMD.

GSDMD residues Leu304, Leu308, Val364, and Leu367 are documented to form hydrophobic contacts with Caspase-1 βIII/βIII′ (Figure 1), confirming that the observed labeling reduction at tryptic GSDMD peptide 300–306 was due to masking effects associated with the GSDMD-Caspase-1 exosite interaction.^27^ CID MS/MS of the labeled GSDMD peptide ELELLDR revealed distinct masking effects at Glu300-Leu301, Leu303, and Leu304, reinforcing the suspected binding interactions around this region with amino acid residue-level resolution (Figure S15B). Congruently, the Glu-C GSDMD peptide 301–307 also displayed a masking event, corroborating our findings from the tryptic digest, further suggesting that Leu304 was masked by the interaction with Caspase-1 at the exosite (Figure 2). Indeed, MS/MS analysis showed a single specific masking event at this residue (Figure S17B). No labeling differences were observed on GSDMD peptide 308–321; however, interrogation of the crystal structure showed that this peptide (excluding Leu308) was located away from the exosite, suggesting that any labeling differences would be largely hidden by neighboring residues with high chemical accessibility and therefore difficult to detect.^27^ Further indication of exosite interaction was demonstrated by masked Glu-C GSDMD peptides. Peptide 355–366 contained Val364, which was known to make contacts with Caspase-1, and it appeared logical that the labeling reduction observed on this peptide reflected binding interactions involving the residue. MS/MS of labeled CLVLSSGMLVPE revealed that residues 355–361 (CLVLSSG) were labeled and remaining 361–366 (MLVPE) were unlabeled (Figure S17B), hence locating masking effects to the N-terminal half of the peptide. Interrogation of the CTD-GSDMD/Caspase-1 crystal structure revealed that GSDMD peptide 355–361 was extremely proximal to caspase-1 βIII/βIII′, and despite these residues not directly contacting the heterodimer, masking was presumably due to blocking by bound caspase-1.^27^ GSDMD peptides 332–354 or 335–354 did not contain any residues that were known to form direct contacts with Caspase-1. MS/MS showed that residues 332–342 were unlabeled whilst carbene modification was found on residues 343–354, forming a contiguous region of masking with the following peptide, 355–361 (Figures 2 and S17B). It should be borne in mind that the size of NaTDB (approximately 8.5 Å in length) may result in masking effects over a greater area than the exact contact surface, which may reflect the observed results. However, in keeping with our observations at peptide 355–361, it appeared that the masking effects on 343–354 represented similar steric protection caused by Caspase-1 βIII′ proximity, again reiterating exosite interaction between GSDMD and the cysteine protease (Figure 2).

On the Caspase-1 p20 subunit, masking events were observed at peptides 279–286, 287–296, and 287–297 (Figures 2 and S19–S22). Two significant labeling reductions were also seen on the p10 subunit of hCaspase-1, at peptides 342–352 and 375–383 (Figures 2 and S19–S23). Masking on the p20 subunit mapped to βIII and the L2 loop, whilst labeling reduction on p20 peptides 287–296 and 287–297 provided further evidence for GSDMD/Caspase-1 binding at the exosite region, in accordance with GSDMD footprinting data. MS/MS analysis of p20 peptide 287–297 revealed residue-level labeling, with specific reductions at Trp294, Val293, and Val292 (Figures 2 and S20B). Given the role that Trp294 plays in binding to the hydrophobic groove of GSDMD,^27^ labeling reductions were attributed to the formation of these contacts, highlighting the power that carbene footprinting and specifically MS/MS-based approaches have in identifying high-resolution interaction sites. Due to low sequence coverage and lack of labeling on the NTD of the Caspase-1 p10 subunit, we were not able to detect the binding of βIII′ to the hydrophobic groove of GSDMD.

Attention was next turned to the catalytic domain of Caspase-1 and, specifically, whether its interaction with the GSDMD linker region (containing the cleavage site) could be detected by carbene footprinting. Whilst these interactions remain unknown, it was anticipated that hGSDMD/hCaspase-1 would make analogous binding contacts to those of the mGSDMD/hCaspase-1 structure (Figure 1).^23^ Unfortunately, masking effects were not observed on the GSDMD linker peptide 268–291. This peptide contained the tetrapeptide FLTD and so was anticipated to contact the Caspase-1 active site. MS/MS of the labeled GSDMD peptide 268–291 showed that carbene modification was located between regions 268–277 and 278–291 (Figure S15B). The former region contained the (FLT)D cleavage site and exhibited a three-fold reduction in labeling in the presence of Caspase-1, but this difference was not statistically significant perhaps due to structural flexibility of the loop. Returning to Caspase-1 p20, peptide 279–286 constituted much of the Caspase-1 catalytic site and the observed masking event at this peptide evidenced its interaction with the linker region. Indeed, further MS/MS analysis revealed carbene modification to residues 279–283 but not residues 284–286 (Figures 2 and S20B), meaning that the masking observed was confined to the 5 N-terminal residues. Since the C285A Caspase-1 construct was employed in this study to prevent catalytic turnover of GSDMD, interaction of _Casp_Ala285 with the linker peptide was not anticipated. However, _Casp_Gln283 is known to form side-chain hydrogen bonds to _Gas_Asp276, and the observed masking events on Caspase-1 peptide VIIIQ supported the notion of GSDMD linker binding to the catalytic domain of Caspase-1. Therefore, despite being unable to detect masking on the hGSDMD linker directly, due to a lack of labeling, differential study of both proteins allowed characterization of active site-based interactions on Caspase-1.

For the Caspase-1 p10 subunit, significant reductions in labeling were seen at peptides 342–352 and 375–383. Much of the region around peptide 342–352 is known to contact the mGSDMD linker. For example, _Casp_Arg341, Trp340, and Pro343 are in proximity of _Gas_Leu274 and Ser275 whilst _Gas_Leu273 and Ser272 are also reported to make van der Waals and hydrophobic contacts with _Casp_Arg383, Trp340, and His342.^23^ The masking effects observed on Caspase-1 peptide 342–352 support linker binding on and around this region of Caspase-1. Indeed, MS/MS showed residue-level masking events at His342, Met345, and Gly346, with very low/no labeling observed at Pro343 (Figures 2 and S21B). Therefore, given the known role that this region, and indeed, _Casp_His342 play in mGSDMD linker binding, we were able to again report binding to the hCaspase-1 (C285A) catalytic domain. This was also reinforced by peptide-level masking on Caspase-1 peptide 375–383, given Arg383’s role in contacting mGSDMD. MS/MS showed carbene modification to residues 375–379, with labeling differences occurring on all labeled residues. Whilst labeling did not occur on Arg383, masking events on nearby residues again reflected proximity to the hGSDMD linker (Figures 2 and S21B).

### 2.3 Carbene Labeling of NTD-GSDMD in Liposomes

**Figure 3:**
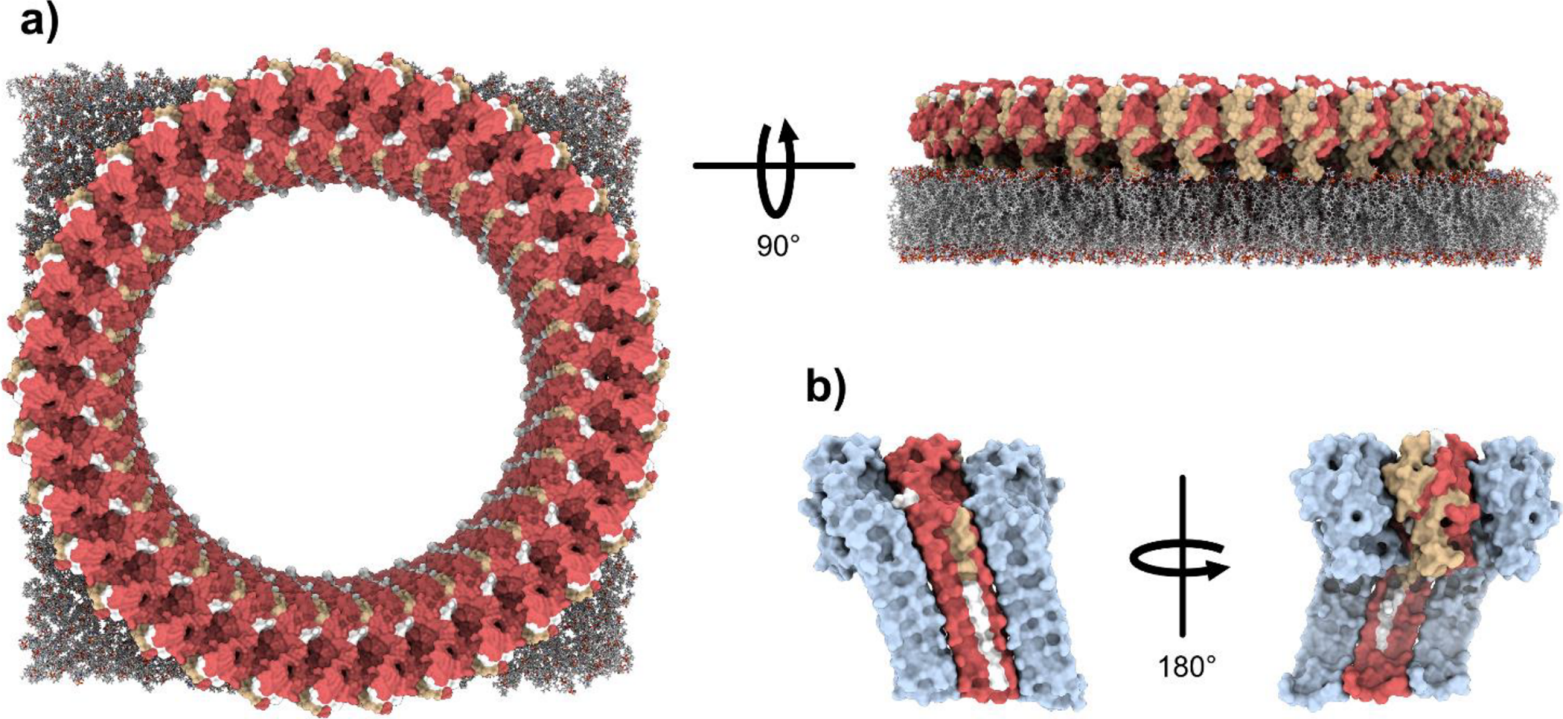
Relative change in fractional modification between full-length hGSDMD monomers and hGSDMD-NT pores. Labeling data were mapped onto (a) the lipid membrane-embedded pore. This was modeled with the CHARMM-GUI bilayer builder using a POPC:CL (1:1) membrane and PDB 6VFE. Color scheme: red = masking, tan = no change, gray = no labeling, white = no peptide coverage. (b) Trimer sub-structure showing the same color scheme on the central hGSDMD-NT only for clarity.

The N-terminal domain (NTD) of hGSDMD in pores generated from 1-palmitoyl-2-oleoyl-sn-glycero-3-phosphocholine (POPC) and 1′,3′-bis[1,2-dioleoyl-sn-glycero-3-phospho]-glycerol(CL) was labeled to identify changes associated with protein oligomerization and lipid binding. The relative change in fractional modification was compared between full-length monomer hGSDMD and the oligomerized NTD pore version (Figure S24). Most tryptic peptides displayed a reduction in carbene labeling compared to full-length GSDMD; however, several regions showed no change in fractional modification between the two species. Labeling reductions were mapped on the NTD-GSDMD pore subunits, which revealed extensive masking effects on membrane-spanning β-sheets and oligomerization contact surfaces (Figures 3 and S24). Several relative masking events were observed on seemingly exposed regions; however, these constituted long tryptic peptides which also partly contacted interfacial sites or the lipid bilayer. This masking likely reflected the combined steric effects of pore formation and lipid-insertion, compared to labeling of the more accessible soluble monomer. Nonetheless, peptides on the solvent-accessible lip portion of GSDMD-NT primarily displayed no change in carbene modification compared to the full-length monomer, suggesting no change in chemical accessibility of these regions. Taken together, the observed changes were consistent with monomer self-assembly and pore formation and highlight the versatility of using carbene footprinting to identify gross structural changes of lipid-embedded protein assemblies, which are challenging targets for structural study.

### 2.4 Differential Footprinting of Caspase-1/VRT-043198

Carbene labeling was next applied to Caspase-1 in the presence and absence of VRT-043198 (the active metabolite of VX-765), as described in the Materials and Methods section. This compound normally reacts with the active site Cys285 thiol group of Caspase-1.^25^ The C285A mutant employed in this study was therefore unable to undergo covalent modification. Shape complementarity has been suggested to mediate important binding contacts between VRT-043198 and Caspase-1, which increase the selectivity and overall efficacy of interactions. As such, we sought to determine whether VRT-043198 would bind noncovalently to the protease in the absence of Cys285.

**Figure 4:**
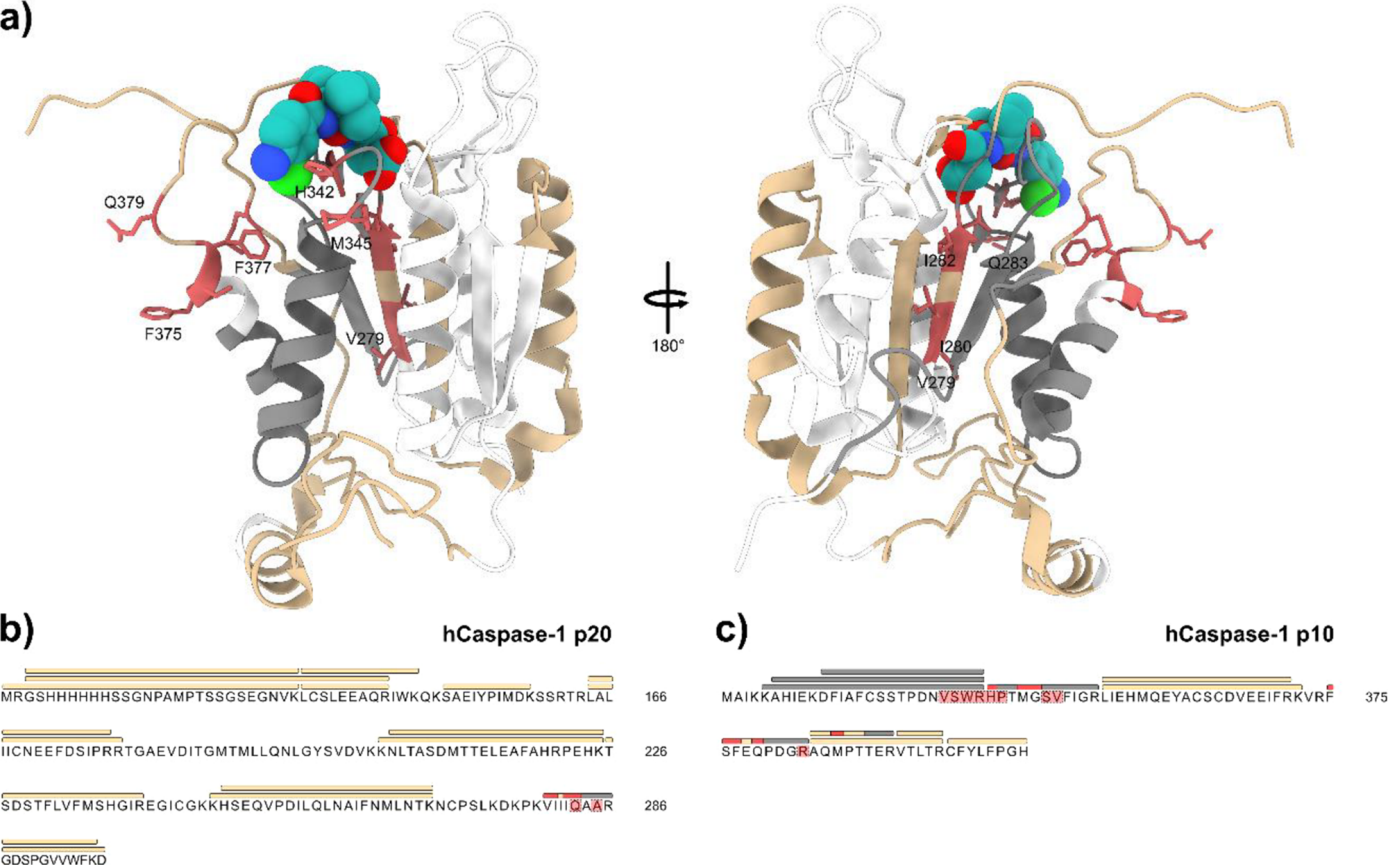
Carbene footprinting of hCaspase-1-VRT-043198. (a) Labeling results mapped onto the hCaspase-1-VRT-043198 complex. Color scheme is as follows: red = masking effect, tan = no change, gray = no labeling, white = no peptide coverage. (b) Labeling results mapped onto the hCaspase-1 p20 sequence. Bars above the sequence represent peptides whilst highlighted residues indicate predicted interaction regions. Coloring is the same as above. (c) Labeling results mapped onto the hCaspase-1 p10 sequence.

The addition of DMSO (1% v/v) to Caspase-1 solution is necessary to solubilize VRT-043198. For the differential study, an equivalent amount of DMSO was added to the control and VRT-043198 treated protein. The addition of VRT-043198 induced a single labeling reduction on the p20 subunit at peptide 279–286 (Figures 4, S25, and S26A) and three significant labeling reductions on the p10 subunit, including at peptides 342–352, 375–383, and 384–391 (Figures 4 and S27A). Pleasingly, when mapped to the Caspase-1/VRT-043198 crystal structure (PDB 6PZP), these masking events localized to the ligand binding site, suggesting that despite the absence of the catalytic residue, the metabolite was binding to Caspase-1 (C285A) in a similar region to that of wildtype Caspase-1.^29^ MS/MS was again utilized to provide higher resolution labeling information on masked peptides. CID fragmentation showed consistent fragmentation patterns, and similar residue-level labeling, to those observed in the GSDMD/Caspase-1 labeling study (see Section 2.2). The active site peptide 279–286 on p20 displayed significant residue-level labeling effects at residues Val279-Ile280, Ile282, and Gln283 (Figures 4 and S26B). Labeling reductions at Gln283 were attributed to hydrogen bond formation with the carboxylic acid of VRT-043198 whilst differences on neighboring residues were likely caused by the proximity of the ligand and consequential steric hindrance to the carbene. On the p10 subunit, Val338 and Trp340 were predicted to form pi-alkyl interactions with the pyrrolidine ring whilst Val348 and Pro343 were suggested to interact with the *tert*-butyl moiety (Figures 4 and S28). Pi–pi interactions were also predicted between His342 and the aromatic ring of the chloroaniline group, as well as hydrogen bond formation between Arg341 and the benzamide moiety and Ser339 and a secondary amide moiety. Ser347 was also anticipated to form van der Waals interactions with the metabolite. The reduction in carbene labeling on peptide 342–352 in the presence of VRT-043198 likely reflected these interactions, with MS/MS revealing significant labeling events on His342, Met345, and Gly346 (Figures 4 and S27B). Peptide-level labeling reductions at 375–383 and 384–391 presumably reflected Arg383’s pi-cation interaction with VRT-043198’s chloroaniliine moiety but also by the overall proximity of these peptides to the compound, causing masking from the photoactive probe. MS/MS displayed significant labeling events on Phe375-Ser383, Phe376, Glu378, and Met386 (Figure S27B). These findings demonstrate a specific noncovalent interaction between VRT-043198 and hCaspase-1 (C285A) and highlight the ability of carbene footprinting methodology to rapidly discern the binding of noncovalent inhibitors in the hCaspase-1 active site.

## 3 Conclusions

In summary, using a dual-protease approach, we have shown that carbene footprinting mass spectrometry accurately maps the exosite and catalytic domain interaction between full-length human GSDMD and human Caspase-1. MS/MS was used to highlight residue-level masking events at interaction sites, consistent with CTD-hGSDMD and full-length-mGSDMD X-ray crystallography structures, but with the benefit of using full length hGSDMD and reporting data on the linker peptide occupancy of the hCaspase-1 active site. The application of carbene footprinting to the pore-forming NTD of GSDMD showed how changes in labeling compared to the full-length protein were consistent with oligomerization. The technique was also used to show noncovalent interaction of VRT-043198 with the protease C285A, in a similar structural arrangement to that of the wildtype. These results demonstrate the feasibility of using carbene footprinting to understand and characterize protein–ligand interactions.

## 4 Materials and Methods

### 4.1 General

Proteases were purchased from Promega (Chilworth, UK). Nano-ESI fused silica tips were purchased from MS WIL (Arle-Rixtel, the Netherlands). 4-(3-Trifluoromethyl)-3H-diazirin-3-yl)benzoic acid (TDBA) was purchased from Novabiochem, (Merck, Dorset, UK). Mini-PROTEAN TGX Precast Protein Gels (12%) (10-well, 30 μL) were purchased from Bio-Rad (Hertfordshire, United Kingdom). PageRuler Prestained Protein Ladder, GelCode Blue Safe Protein Stain, and Snap Ring Micro-Vials (11 mm) were purchased from Thermo Fisher Scientific (Loughborough, UK). The active metabolite of Belnacasan, VRT-043198, was sourced from MedChemExpress (Distributer Insight Biotechnology, Wembley, UK or Cambridge Bioscience, Cambridge, UK). All other chemical and buffers were purchased from Thermo Fisher Scientific or Sigma-Aldrich (Merck, Dorset, UK).

### 4.2 Expression and Purification of GSDMD, GSDMD-NT Liposomes, and Caspase-1

The coding sequence human GSDMD (UniProt: P57764) were synthesized by TWIST bioscience into a bacterial expression vector encoding a His6-SUMO tag and transformed in BL21 (DE3) Gold (Agilent Technologies) for overexpression. 2xYT (Fisher Scientific) cultures were grown at 37 °C until OD600 reached 0.8, and protein expression was induced at 18 °C overnight with 0.5 mM isopropyl β-D-1-thiogalactopyranoside (IPTG). Cells were harvested and lysed by high shear homogenization using a microfluidizer (Analytik) in a lysis buffer containing 25 mM Tris–HCl (pH 8.0), 150 mM NaCl, and 0.5 mM TCEP. The recombinant SUMO-fusion protein in the cleared cell lysate was purified by affinity chromatography using a 5 mL HisTrap affinity column (Cytiva). Eluted fractions containing the enriched SUMO-fusion protein were pooled and dialyzed for 24 h at 4 °C against imidazole-free lysis buffer with the Ulp1 protease (Sigma-Aldrich), followed by anion exchange chromatography (5 mL Q HiTrap HP, Cytiva) to remove the SUMO tag and any uncleaved fusion protein. The samples containing GSDMD were further purified through Superdex 75 (Cytiva) size-exclusion chromatography in a buffer containing 20 mM HEPES, 150 mM NaCl, and 1 mM TCEP at 4 °C. The purified protein at concentrations between 50–100 μM was frozen in aliquots at −80 °C.

CL (18:1) was mixed with POPC (16:0–18:0) (Avanti Polar Lipids) at a molar ratio of 1:3. Lipid cakes generated by evaporating the solvent chloroform were resuspended in buffer containing 20 mM HEPES, 150 mM NaCl by vigorous vortexing and iterative rounds of freeze/thaw cycles. The resulting liposomes were extruded through a 100 nm filter (Whatman) with 35 passes to generate large, unilamellar vesicles, diluted to approximately 1 mM in buffer. Purified GSDMD was added to a final concentration of 2.5 μM, along with 100 U of recombinant Caspase-1 (Enzo). The reaction was allowed to proceed for 30 min at room temperature before pelleting the proteoliposomes by centrifugation at 100,000 × *g* and 4 °C for 30 min. The supernatant containing all soluble remnants (intact full-length GSDMD, CTD, and Caspase-1) was discarded, and the pellets were stored at −80 °C.

The coding sequence for human Caspase-1 p20 and p10 fragments encoding a catalytically inactivating C285A mutation was synthesized by TWIST bioscience into a polycistronic bacterial expression vector encoding an N-terminal His-tag. and transformed in BL21 (DE3) Gold (Agilent Technologies) for overexpression. 2xYT (Fisher Scientific) cultures were grown at 37 °C until OD600 reached 0.8 and protein expression was induced at 18 °C overnight with 0.5 mM isopropyl β-D-1-thiogalactopyranoside (IPTG). Cells were harvested and purified as detailed by Roschitzki-Voser et al.^30^ Briefly, inclusion bodies were isolated from the whole cell lysate and solubilized using GnHCl. Refolding was performed by rapid dilution in a solution containing 100 mM HEPES, 100 mM NaCl, 100 mM sodium malonate, 20% sucrose, 0.5 M NDSB-201, and 10 mM DTT. The cleared resuspension was purified by affinity chromatography (5 mL HisTrap HP), followed by cation exchange (5 mL SP HP HiTrap) and gel filtration (Superdex 75) (Cytiva). The purified p20/p10 dimer was concentrated to 100 μM, aliquoted, and frozen at −80 °C.

### 4.3 Native-PAGE

Caspase-1 (40 μM, 3 μL), GSDMD (40 μM, 3 μL), GSDMD/Caspase-1 (30 μM protein 1:1 equiv, 5 μL), GSDMD/Caspase-1 with water (24 μM protein 1:1 equiv, 5 μL), and GSDMD/Caspase-1 complex with NaTDB (20 mM diazirine, 24 μM protein 1:1 equiv, 5 μL) were combined with an equal volume of 2× native-PAGE loading buffer (40% glycerol, 0.01% Bromophenol Blue, 62.5 mM Tris–HCl, pH 6.8). The same samples but with 2:1 equiv Caspase-1/GSDMD were also prepared. Samples were immediately analyzed with native-PAGE (12% polyacrylamide gel, 150 V) in running buffer (25 mM Tris–HCl, 192 mM glycine, pH 8.3). Gels were stained with GelCode Blue Safe Protein Stain.

### 4.4 SDS-PAGE

Protein samples were combined with 6× Laemmli SDS sample buffer (375 mM Tris–HCl, 9% (w/v) SDS, 50% (v/v) glycerol, 0.03% (w/v) bromophenol blue, 9% (v/v) beta-mercaptoethanol), incubated at 95 °C for 5 min, and analyzed with SDS-PAGE (12% polyacrylamide gel, 150 V) in running buffer (25 mM Tris–HCl, 192 mM glycine, 0.1% SDS, pH 8.3) alongside PageRuler Prestained Protein Ladder. Gels were stained with GelCode Blue Safe Protein Stain.

### 4.5 Proteolysis

To aid in the selection of the most appropriate proteases, in-silico protein digestion was performed on hGSDMD and hcaspase-1 using the PeptideCutter server.^31^

Protein bands were excised from SDS-PAGE gels using a scalpel, cut into 1 mm^2^ pieces, and destained with aqueous acetonitrile (MeCN) solution (50%, 50 μL) for 10 min at room temperature. Gel pieces were dehydrated with acetonitrile (MeCN, 450 μL) with agitating for 3 min before the MeCN was removed. They were then treated with DTT solution (DTT 10 mM, ammonium bicarbonate (AmBic) 100 mM, 50 μL) at 55 °C for 30 min before being dehydrated with MeCN (450 μL). Gel pieces were treated with iodoacetamide solution (iodoacetamide 55 mM, AmBic 100 mM, 50 μL) and incubated in the dark for 30 before being dehydrated with MeCN (450 μL). Finally, gel pieces were incubated with the protease (either trypsin or Glu-C, Promega) solution (10 ng/μL, AmBic 50 mM, 50 μL) at 37 °C for 18 h. Formic acid (1 μL) was added to protein digests to quench protease activity. The supernatant was removed from gel pieces, centrifuged at 5000 × *g* for 5 min, and transferred to plastic 11 mm Snap Ring Micro-Vials for nanoLC-MS analysis.

### 4.6 Liquid Chromatography–Mass Spectrometry

Digests were analyzed with a Dionex U3000 nanoLC coupled to a Thermo Scientific LTQ FT Ultra Mass Spectrometer containing a nano-ESI source. An injection volume of 3 μL was loaded onto a Thermo Scientific C18 Pepmap300 loading column (10 mm, 300 Å, 5 μm particle size). Sample separation was performed using an inline Thermo Scientific C18 Pepmap300 column (150 mm × 75 μm, 300 Å, 5 mm particle size) with a binary solvent gradient of solvent A (5% acetonitrile, 0.1% formic acid) and solvent B (95% acetonitrile, 0.1% formic acid): *t* = 0 min 100% A, *t* = 30 min 45% A, *t* = 30.5 min 10% A, *t* = 35 min 10% A, *t* = 35.5 min 100% A, *t* = 50 min 100% A. The nano-ESI source was operated in positive ion mode with a fused silica emitter nanospray tip (360 μm OD, 10 μm tip ID, 12 cm length) connected to an external voltage supply (1.9 kV) using a liquid–liquid junction.

Protein sequence coverage and peptide identity from both GSDMD and Caspase-1 was determined by operation of the spectrometer in data-dependent acquisition (DDA) mode employing ion trap CID MS/MS of the four most intense precursor peptide ions using helium as a collision gas with a nominal activation energy of 35.0 (and the activation time was set at 30 ms with an activation *Q*-value of 0.250). The precursor isolation window was set to *m/z* 8.0. Database searching was performed using SearchGUI.^32^ Precursor ions were detected in the FTICR cell using a resolving power of 100,000 at *m/z* 400, and MS/MS product ions were detected in the ion trap.

The spectrometer was operated in full scan mode for analysis of carbene labeling. Ions were detected in the FTICR cell using a nominal resolving power setting of 100,000 at *m/z* 400.

### 4.7 Carbene Labeling of GSDMD and Caspase-1

For optimization of diazirine probe concentration, hGSDMD and hCaspase-1 (40 μM, 2.5 μL) were incubated separately for 10 min on ice with an equal volume of NaTBA (either 20, 40, or 80 mM). Aliquots were transferred to plastic 11 mm Snap Ring Micro-Vials and flash-frozen with liquid nitrogen immediately prior to irradiation. Samples were irradiated for 15 s using a Spectra Physics Explorer 349 laser (actively Q-switched Nd:YLF laser 349 nm wavelength, 1000 Hz repetition frequency, 125 μJ pulsed energy) that was vertically reflected into the top of the open vials by a 45° mirror.

Differential hGSDMD-hCaspase-1 binding study. For labeling of bound Caspase-1, excess GSDMD (151 μM, 5.6 μL) was added to caspase-1 (40 μM, 11.2 μL) whilst for labeling of GSDMD, excess caspase-1 (151 μM, 5.6 μL) was added to GSDMD (40 μM, 11.2 μL). Samples (including controls of each separate protein at the same final concentrations) were incubated on ice for 2 h. An aqueous solution of the sodium salt of TDBA (100 mM, 4.2 μL), prepared as described previously,^8^ was combined with either ligand-treated or control samples (GSDMD alone and Caspase-1 buffer, or Caspase-1 alone and GSDMD buffer) to give a final diazirine probe concentration of 20 mM NaTBA. Samples were incubated for a further 10 min on ice. Aliquots (4 replicates of 5 μL) were transferred to plastic 11 mm Snap Ring Micro-Vials and flash-frozen with liquid nitrogen for irradiation as described above.

Carbene labeling of GSDMD-NT pore liposomes. GSDMD-NT pores in POPC:CL (1:1) liposomes (0.1 nmole GSDMD-NT pore, 30 μL) were resuspended with NaTDB (100 mM, 90 μL). Samples were incubated for a further 10 min on ice. Aliquots (4 replicates of 5 μL) were transferred to plastic 11 mm Snap Ring Micro-Vials and flash-frozen with liquid nitrogen for irradiation as described above.

Differential VRT-043198-hCaspase-1 binding study. Excess VRT-043198 (1 mM, 2 μL) in 10% DMSO, stored under N_2_, was added to caspase-1 (40 μM, 18 μL). Samples (including a DMSO-treated control of Caspase-1 alone) were incubated on ice for 30 min. An aqueous solution of the sodium salt of TDBA (100 mM, 5 μL) was combined with ligand-treated and control samples to give a final diazirine probe concentration of 20 mM NaTDB. Samples were incubated for a further 10 min on ice. Aliquots (4 replicates of 5 μL) were transferred to plastic 11 mm Snap Ring Micro-Vials and flash-frozen with liquid nitrogen for irradiation as described above.

### 4.8 Data Analysis of Carbene Labeling

Quantification of carbene labeling was carried out with PepFoot software^33^ using data files acquired in full scan mode. Fractional modifications were generated for peptides from relevant peak areas of unlabeled and labeled extracted ion chromatograms (eq 1).
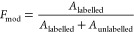
1

For labeling comparisons between hGSDMD monomers and hGSMD-NT pore liposomes, the relative change in fractional modification was determined (eq 2). Propagated standard deviations were also determined.
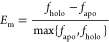
2

Targeted MS/MS was carried out on selected carbene-labeled peptides using manual selection of the appropriate precursor ions. Scans for each labeled peptide were combined across the chromatographic peak(s) to give an average spectrum containing labeled and unlabeled fragment ions from which subpeptide fmods were determined (eq 3).
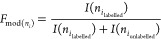
3

Fractional modifications were plotted against digested peptides between control and binding partner-treated samples (if appropriate). Significant labeling differences were determined (*P* < 0.01) and labeling data were mapped onto protein structures.

### 4.9 Homology Modeling of Protein Structure

The full-length hGSDMD structure was modeled with the iTasser Server using 6KN0 as a template.^34^ The generated structure was aligned to PDB 6VIE using the SWISS-MODEL Server.^35^ Carbene labeling results were mapped to this model. Protein structures were visualized in UCSF Chimera.

## Supporting Information Available

In-silico protease coverage; DDA MS/MS peptide sequence coverage; carbene label optimization; native-PAGE binding assays; SDS-PAGE gels; and differential footprinting experiments (PDF)

## Notes

Mass spectrometry raw data have been deposited to the Proteome Xchange Consortium (http://proteomecentral.proteomexchange.org) via the PRIDE partner repository^36^ with the dataset identifier PXD040212.

## Abbreviations

CL: Cardiolipin
CID: Collision-Induced Dissociation
CTD: C-Terminal Domain
ESI: Electrospray Ionization
FPOP: Fast Photochemical Oxidation Of Proteins
GSDMD: Gasdermin D
HDX: Hydrogen-Deuterium Exchange
LC: Liquid Chromatography
MS: Mass Spectrometry
MeCN: Acetonitrile
NaTDB: 4-[3-(trifluoromethyl)-3H-diazirin-3-yl]benzoate
NMR: Nuclear Magnetic Resonance
NTD: N-Terminal Domain
POPC: Phosphocholine
VRT-043198: O-desethyl-belnacasan
VX-765: Belnacasan
