# Supplementary material for: Mapping the Binding Interactions between Human Gasdermin D and Human Caspase-1 Using Carbene Footprinting
Source: JACS Au. 2023 Jun 23;3(7):2025–35. doi: 10.1021/jacsau.3c00236 (PMC10369405; doi:10.1021/jacsau.3c00236)
Supplement: Supplementary file 1 — au3c00236_si_001.pdf [file au3c00236_si_001.pdf]

Mapping the Binding Interactions Between human Gasdermin D and human Caspase-1 Using Carbene Footprinting

James R. Lloyd[§], Antonio Biasutto[+], Katharina L. Dürr[+], Ali Jazayeri[+], Jonathan T.S. Hopper[+*], and Neil J. Oldham[§*]

§ School of Chemistry, University of Nottingham, University Park, Nottingham, NG7 2RD, UK

+ OMass Therapeutics, Schrodinger Building, Oxford Science Park, Oxford, OX4 4GE, UK

KEYWORDS: *carbene labeling, diazirines, protein footprinting, gasdermin D, caspase-1, pyroptosis*

Corresponding Authors: neil.oldham@nottingham.ac.uk, jonathan.hopper@omass.com

SUPPORTING INFORMATION

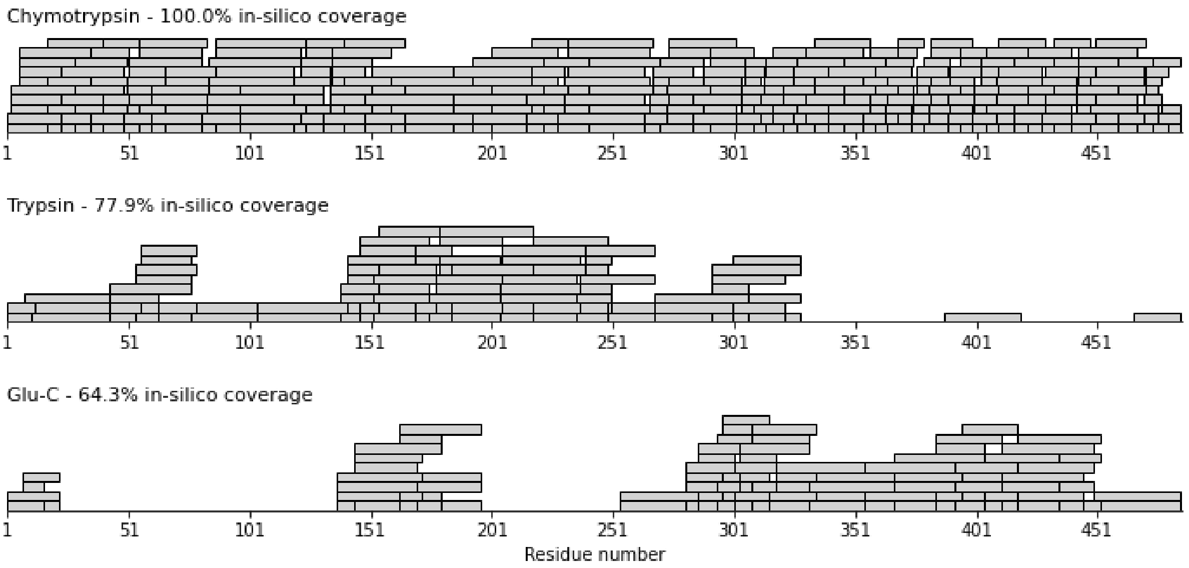

Figure S1. *In-Silico* digest of hGSDMD using the PeptideCutter server with chymotrypsin, trypsin and Glu-C with three missed cleavages. Grey bars represent predicted peptides and overlapping bars indicate missed cleavages.

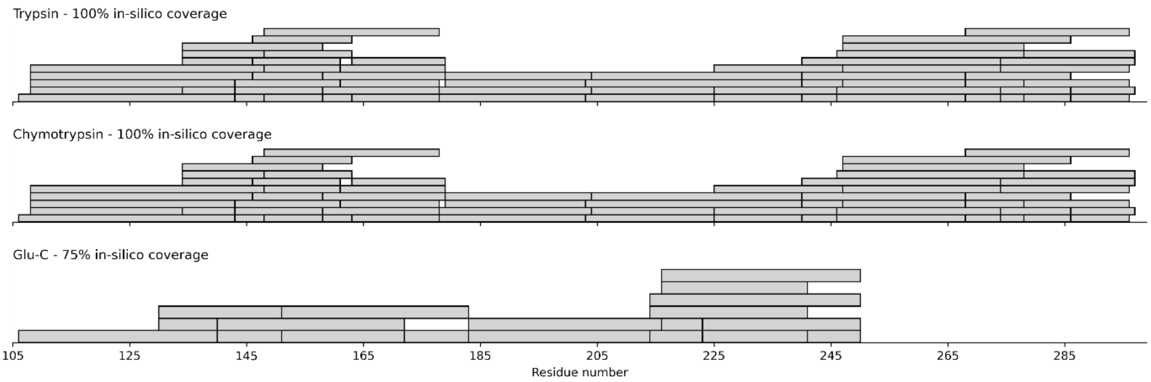

Figure S2. *In-Silico* digest of the Caspase-1 p20 subunit using the PeptideCutter server with trypsin, chymotrypsin and Glu-C with three missed cleavages. Grey bars represent predicted peptides and overlapping bars indicate missed cleavages.

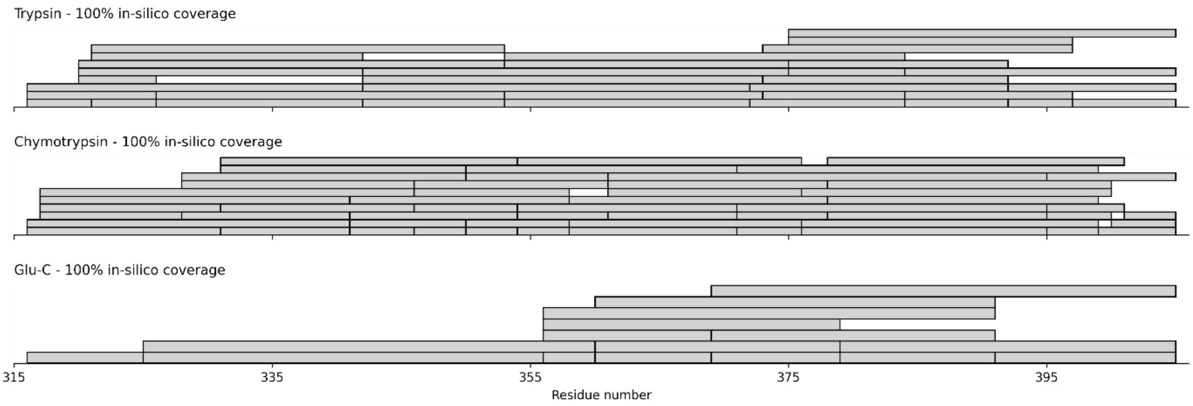

Figure S3. *In-Silico* digest of the Caspase-1 p10 subunit using the PeptideCutter server with trypsin, chymotrypsin and Glu-C with three missed cleavages. Grey bars represent predicted peptides and overlapping bars indicate missed cleavages.

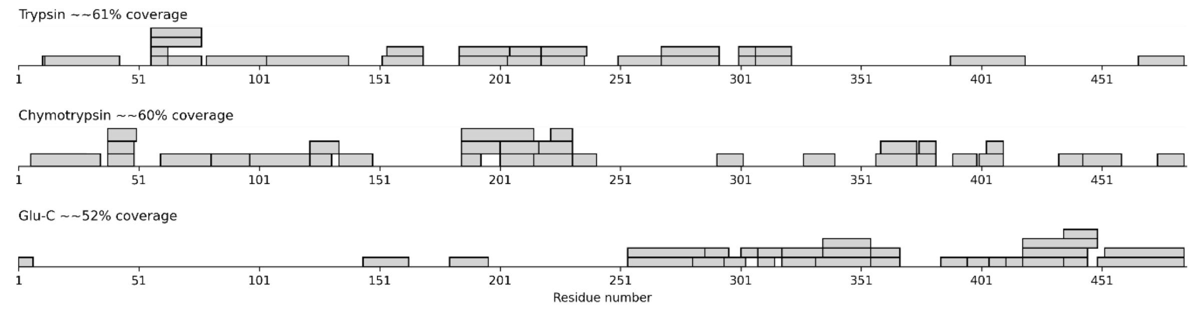

Figure S4. Achieved sequence coverage by DDA LC-MS/MS analysis of hGSDMD following digestion with trypsin, chymotrypsin and Glu-C. Grey bars represent DDA-identified peptides and overlapping bars indicate missed cleavages.

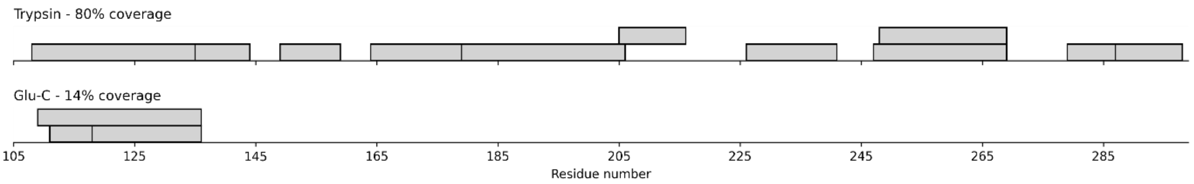

Figure S5. Achieved sequence coverage by DDA LC-MS/MS analysis of the Caspase-1 p20 subunit following digestion with trypsin, chymotrypsin and Glu-C. Grey bars represent DDA-identified peptides and overlapping bars indicate missed cleavages.

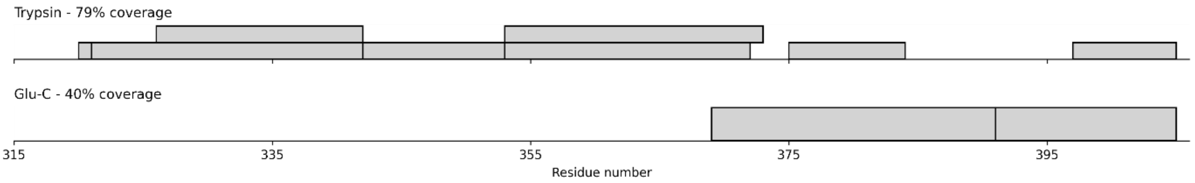

Figure S6. Achieved sequence coverage by DDA LC-MS/MS analysis of the Caspase-1 p10 subunit following digestion with trypsin, chymotrypsin and Glu-C. Grey bars represent DDA-identified peptides and overlapping bars indicate missed cleavages.

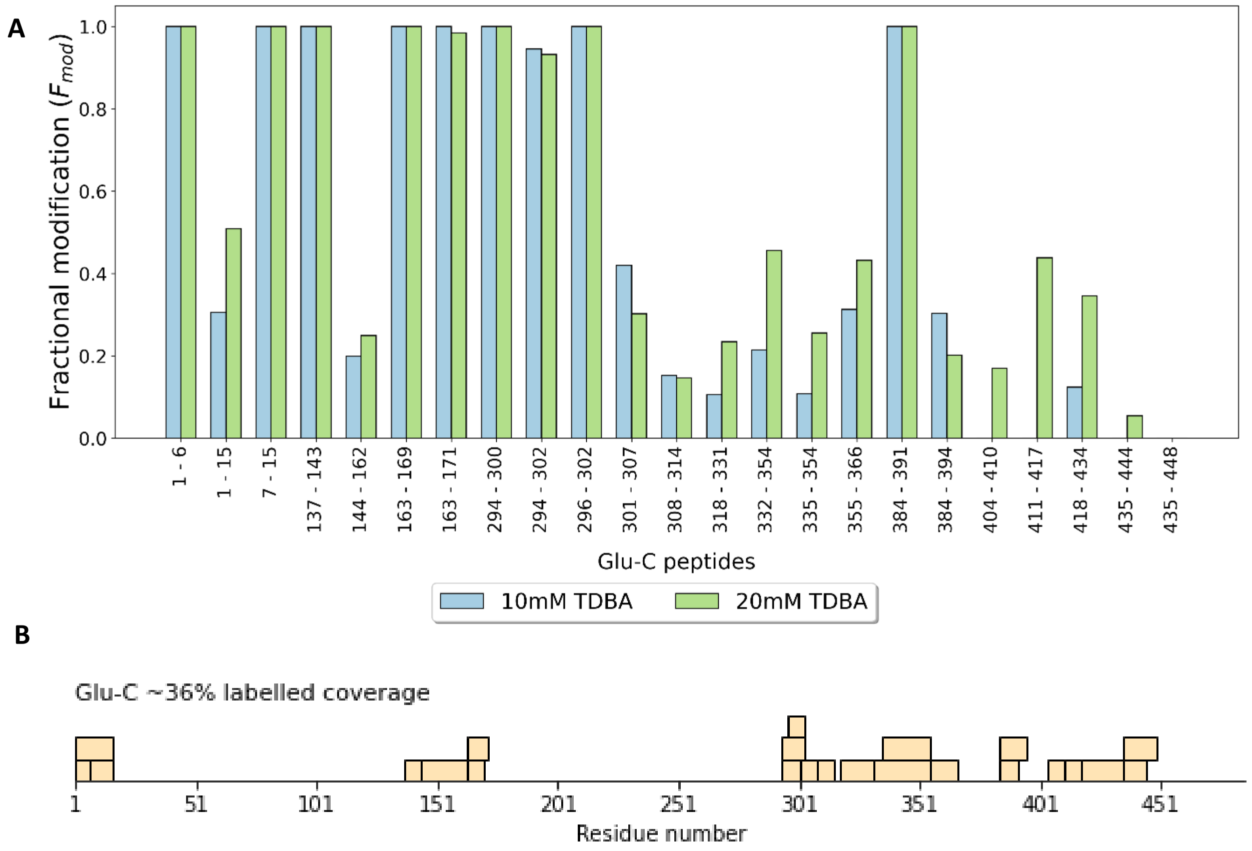

Figure S7. A) Fractional modification of hGSDMD Glu-C peptides at 10 and 20 mM NaTDB showing more efficient labeling at the latter concentration. B) Sequence coverage plot of carbene labeled hGSDMD Glu-C peptides. Oranges bars represent observed peptides and overlapping bars represent missed cleavages.

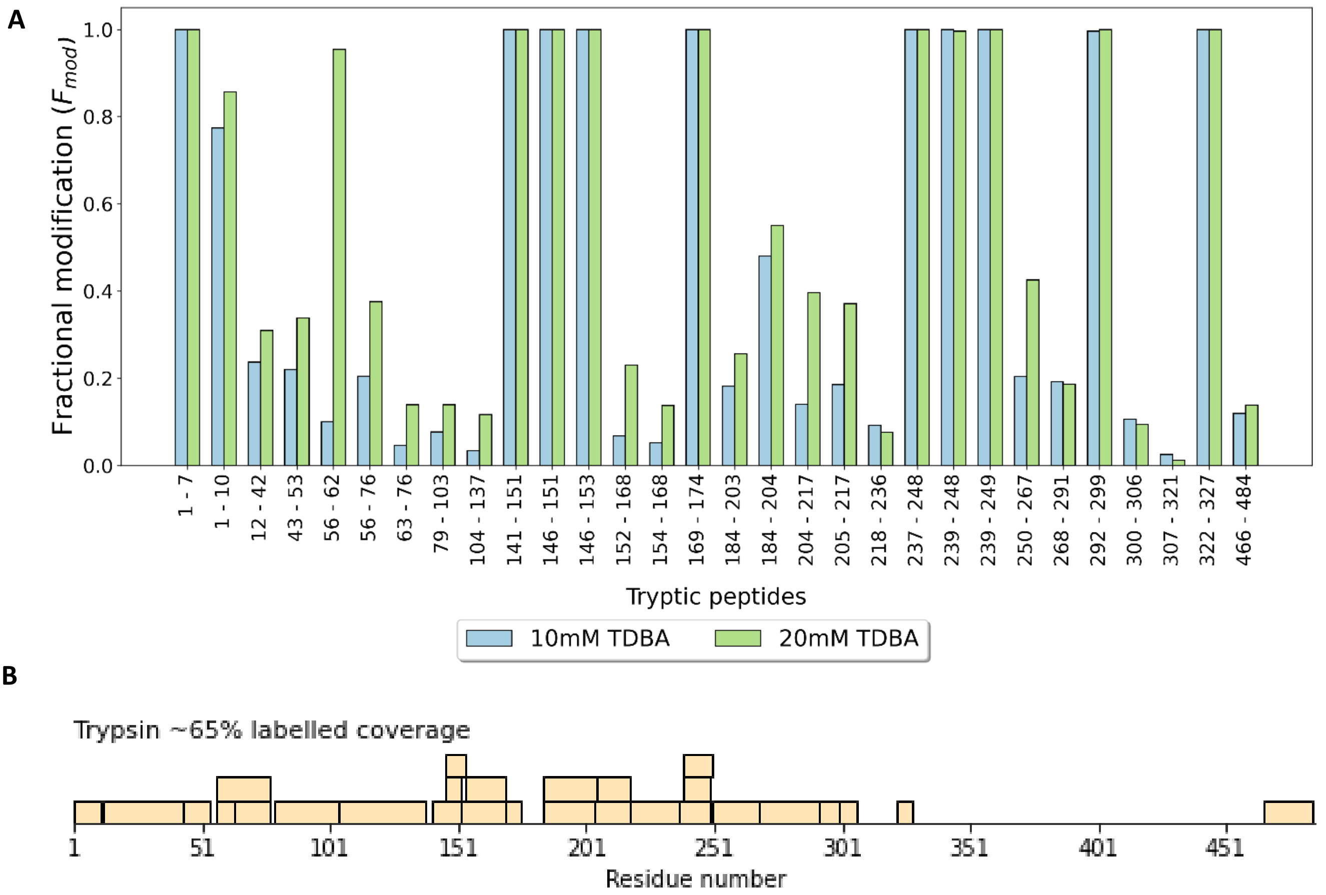

Figure S8. A) Fractional modification of hGSDMD tryptic peptides at 10 and 20 mM NaTDB showing more efficient labeling at the latter concentration. B) Sequence coverage plot of carbene labeled hGSDMD tryptic peptides. Oranges bars represent observed peptides and overlapping bars represent missed cleavages.

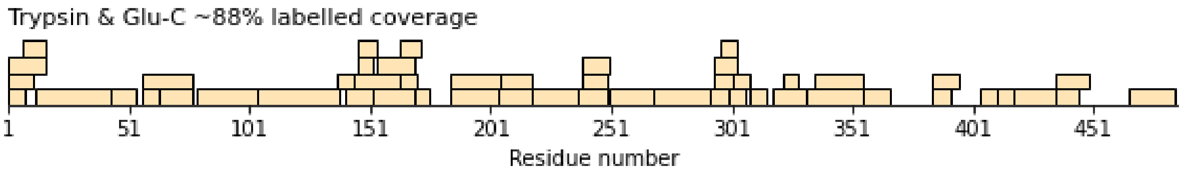

Figure S9. Sequence coverage plot of concatenated carbene labeled hGSDMD Glu-C and tryptic peptides showing overall labeling coverage. Oranges bars represent observed peptides and overlapping bars represent missed cleavages.

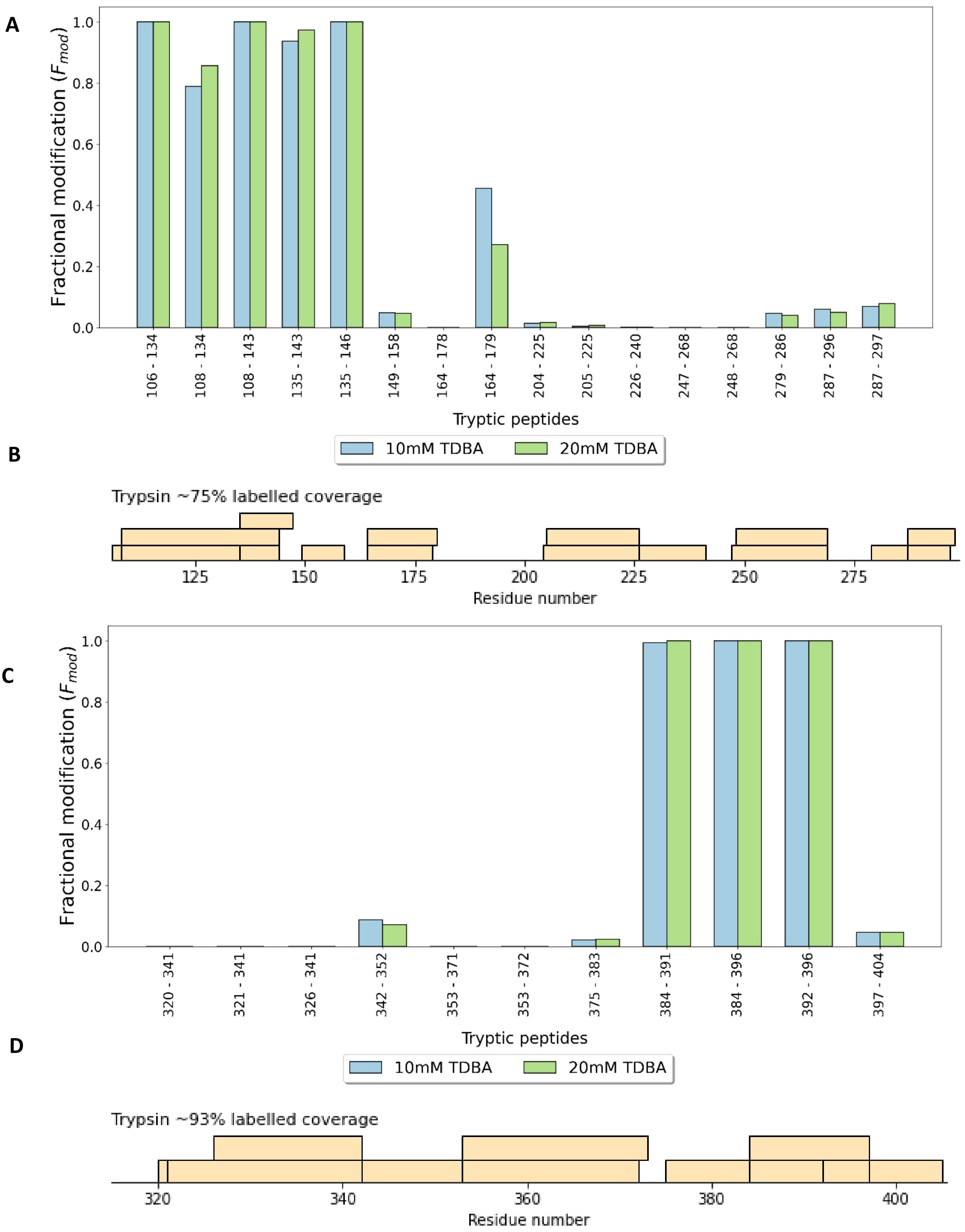

Figure S10. A) Fractional modification of Caspase-1 p10 tryptic peptides at 10 and 20 mM NaTDB. B) Sequence coverage plot of labeled Caspase-1 p10 tryptic peptides. Oranges bars represent observed peptides and overlapping bars represent missed cleavages. C) Fractional modification of Caspase-1 p20 tryptic peptides at 10 and 20 mM NaTDB. D) Sequence coverage plot of labelled Caspase-1 p20 tryptic peptides. Oranges bars represent observed peptides and overlapping bars represent missed cleavages.

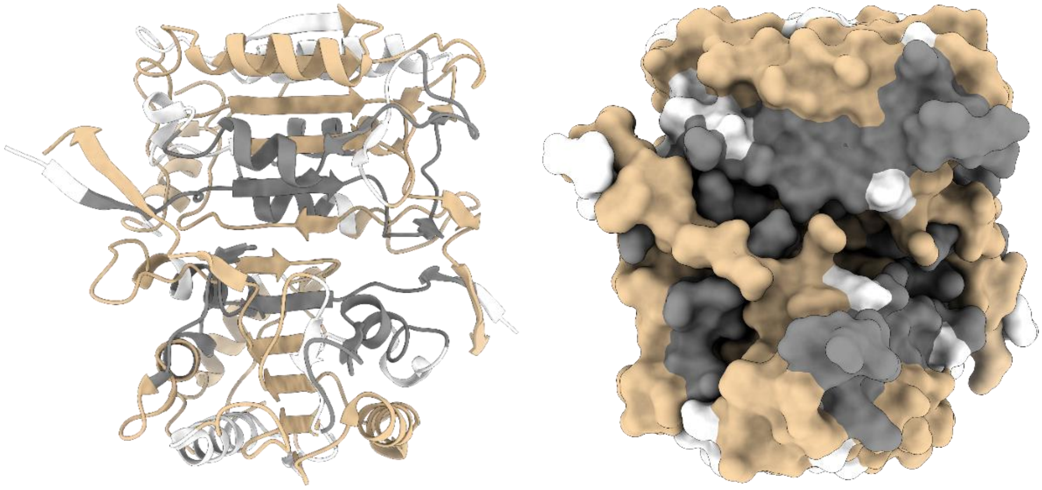

Figure S11. Caspase-1 carbene modification mapped onto the dimer cartoon and surface structure (tan = carbene labeling, grey = no carbene labeling, white = no peptide coverage) showing that the absence of labeling is concentrated at the dimer interface.

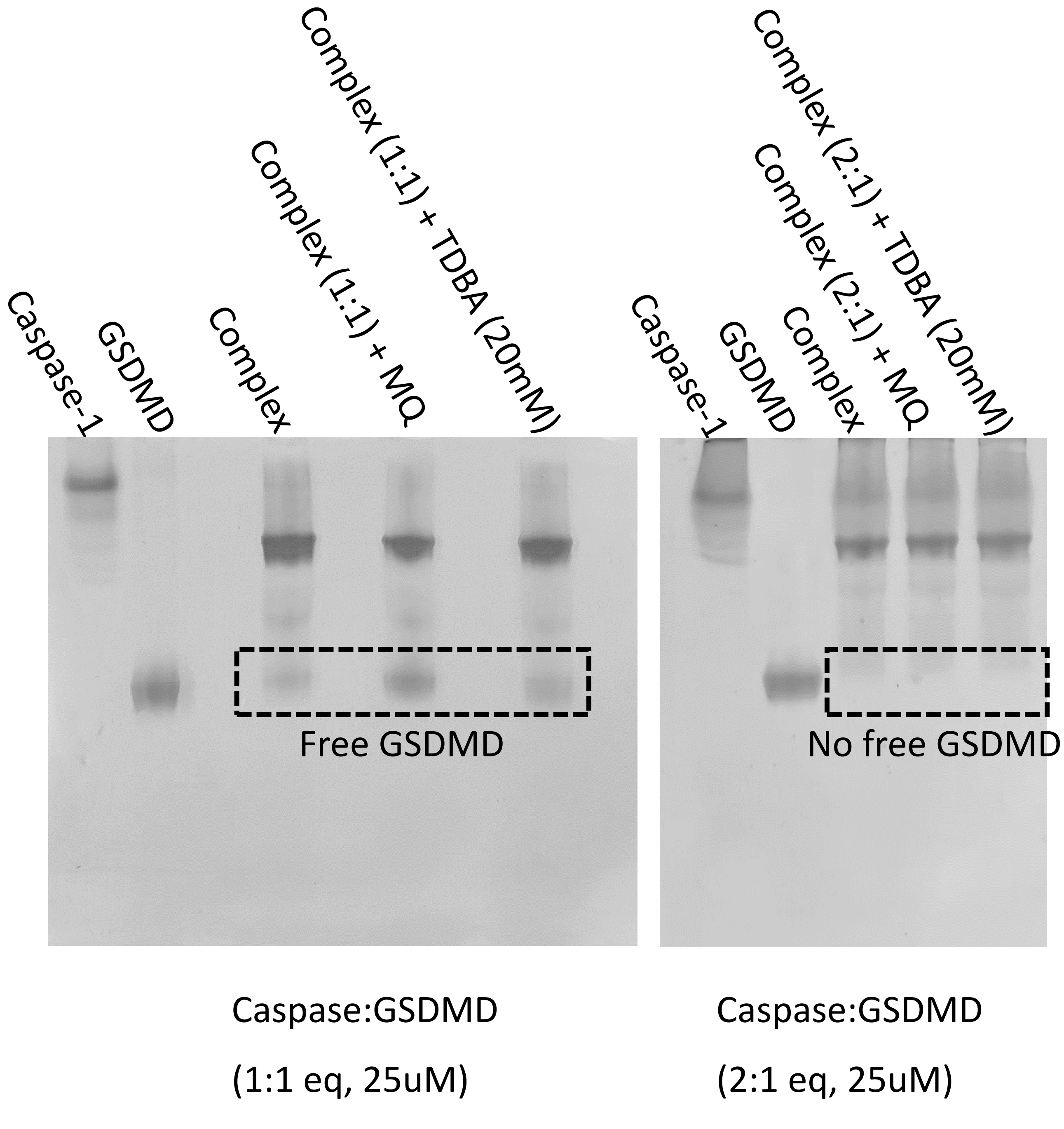

Figure S12. Native-PAGE of the Caspase-1:GSDMD complex at 1:1 and 2:1 equimolar ratios showing that no free GSDMD is present with 2 equivalents of Caspase-1 added.

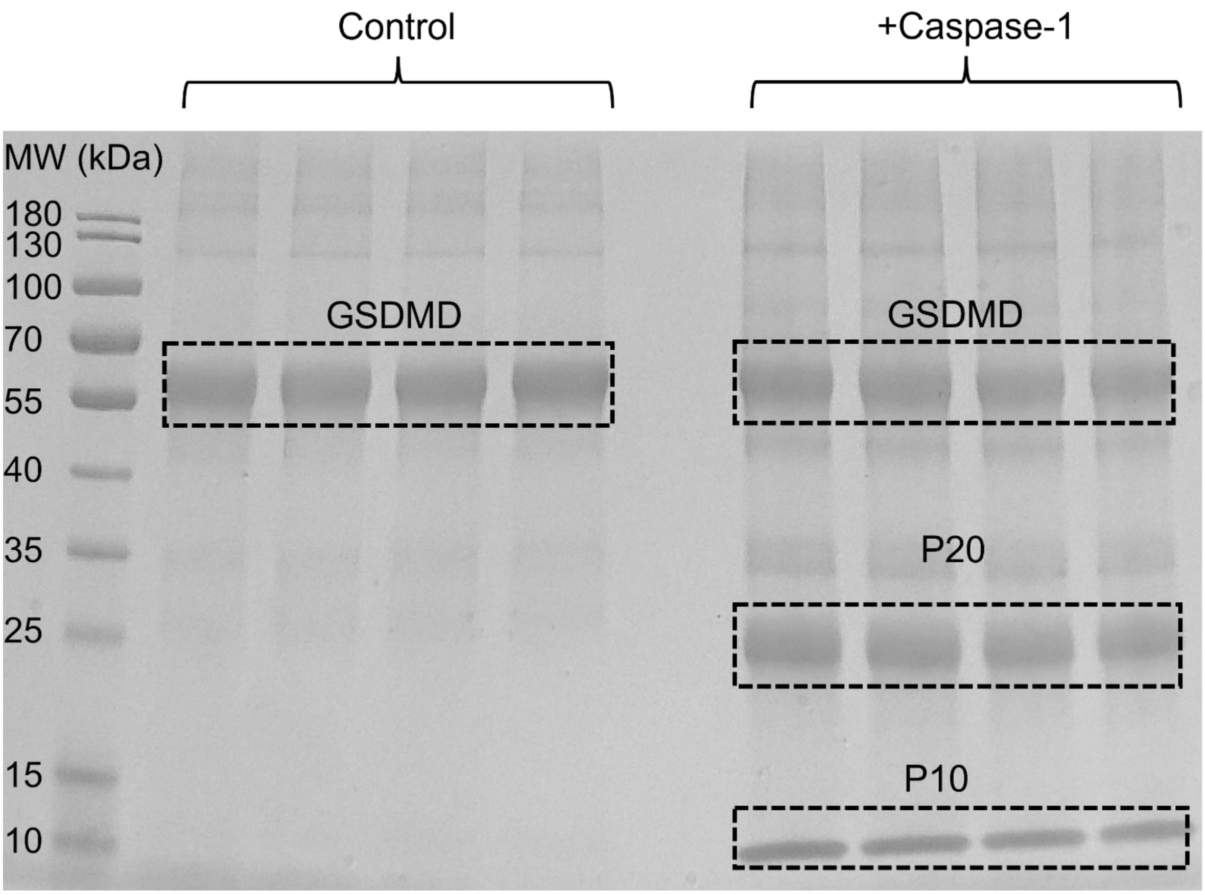

Figure S13. SDS-PAGE of labelled (20 mM NaTDB) GSDMD in the absence (left) and presence (right) of 2:1 molar equivalents of caspase-1 (n = 4). Bands corresponding to the two Caspase-1 subunits, P10 and P20 are labeled.

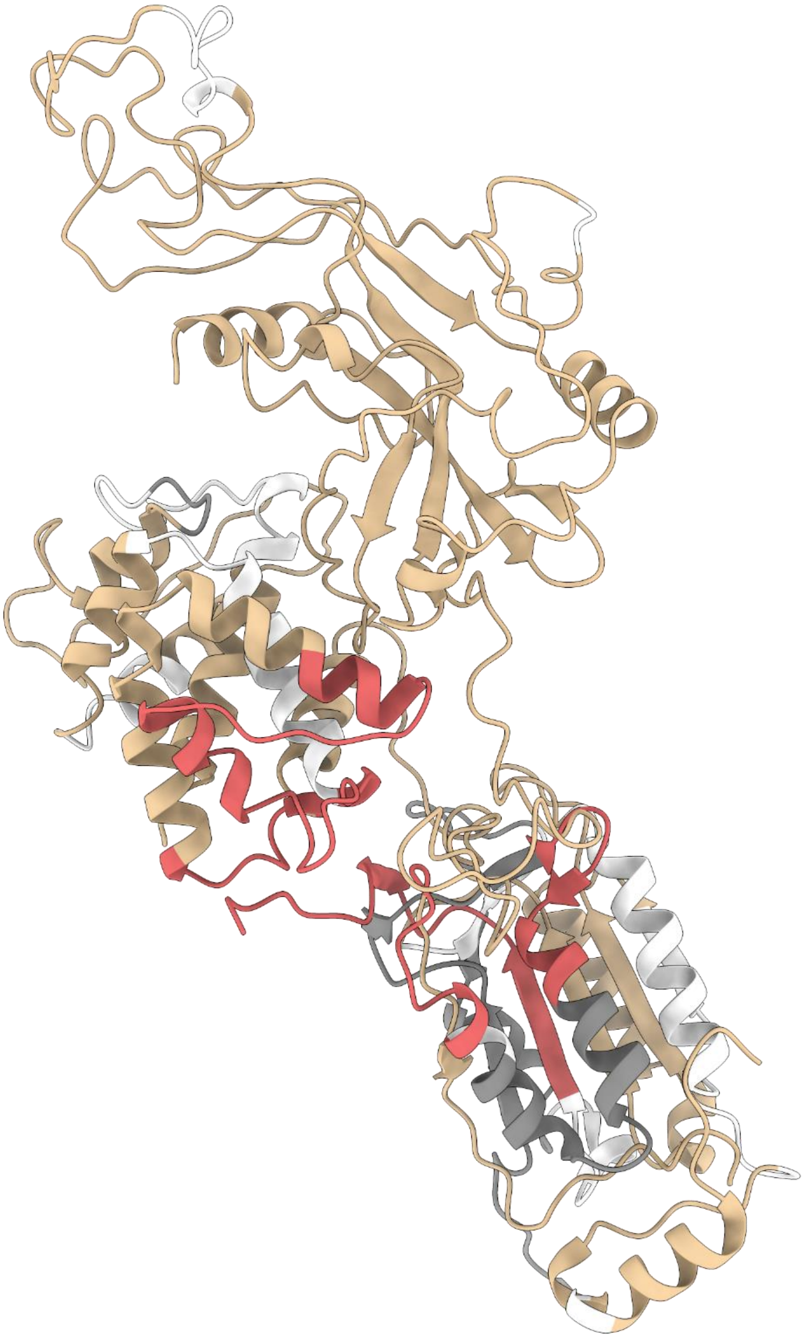

Figure S14. Peptide-level carbene footprinting data mapped onto the hGSDMD:hCaspase-1 structure (tan = carbene labeling, grey = no carbene labeling, white = no peptide coverage, red = reductions in labeling).

A

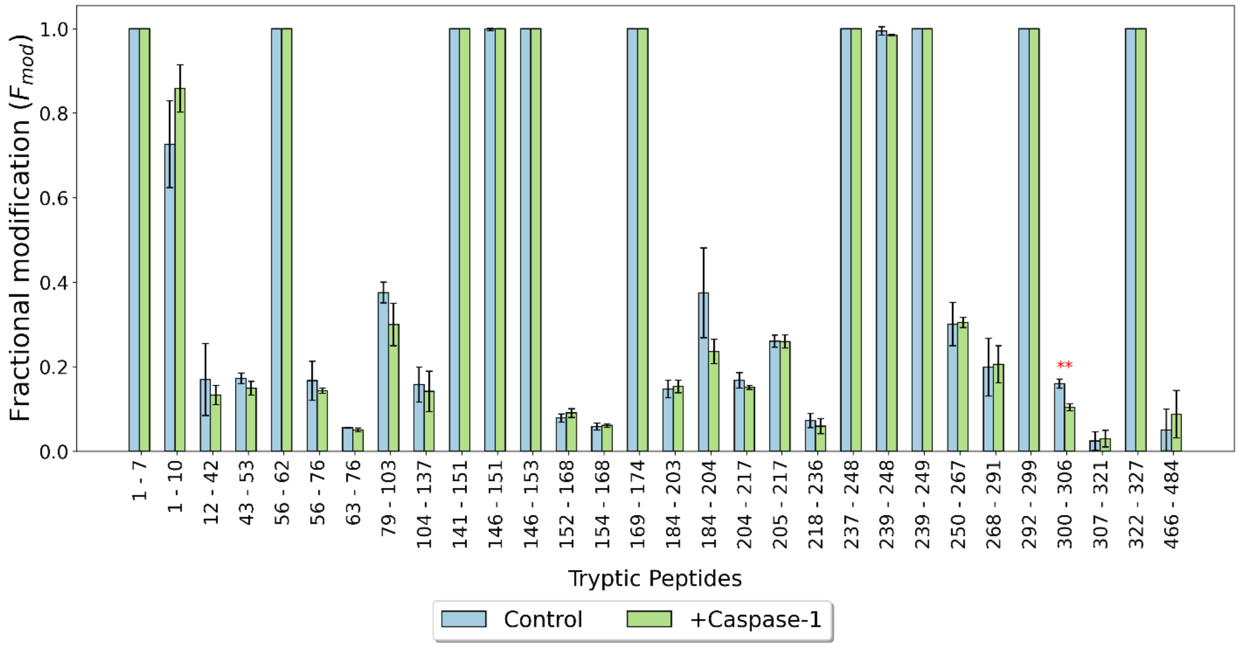

B

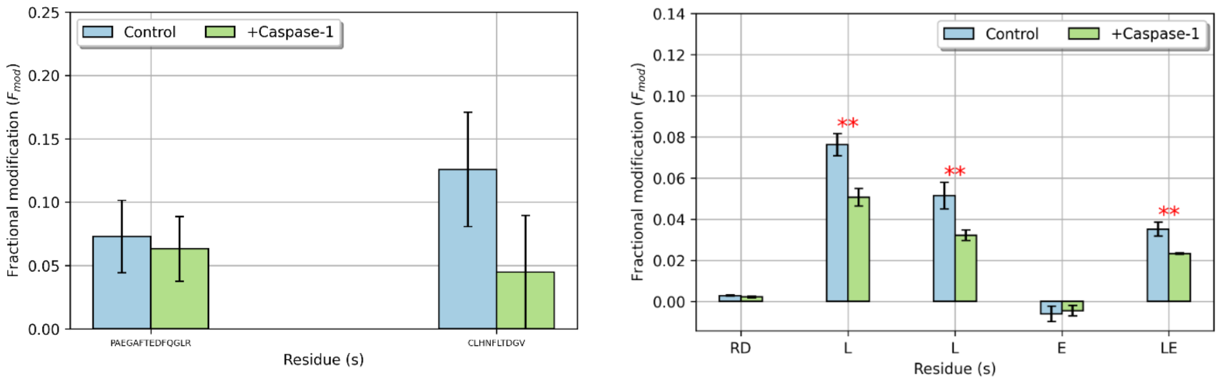

Figure S15. A) Fractional modification of tryptic GSDMD peptides for the differential labeling study, with (green) and without (blue) 2:1 molar equivalents Caspase-1 added. Error bars are ± standard deviation (n = 4). Significant difference between samples is highlighted with ** (Student t-test, P < 0.01). B) Fractional modification of labeled peptides 268-291 (CLHNFLTDGVPAEGAFTEDFQGLR) and 300 – 306 (ELELLDR) following MS/MS fragmentation to reveal sub-peptide level localization. Error bars are ± standard deviation (n = 4). Significant difference between samples is highlighted with * (Student t-test, P < 0.05) or ** (Student t-test, P < 0.01).

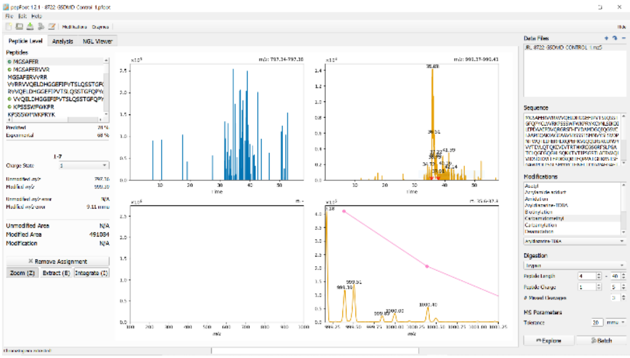
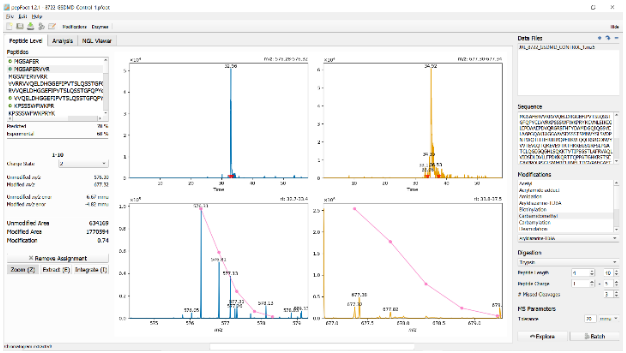
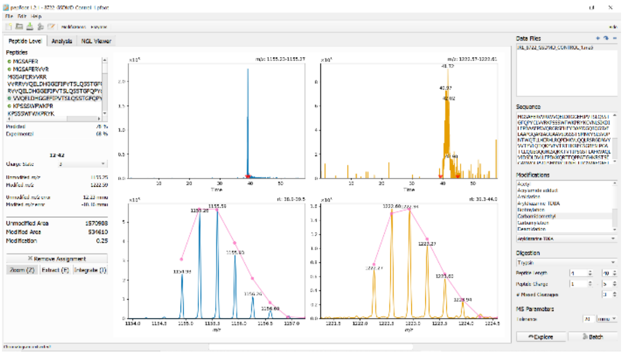
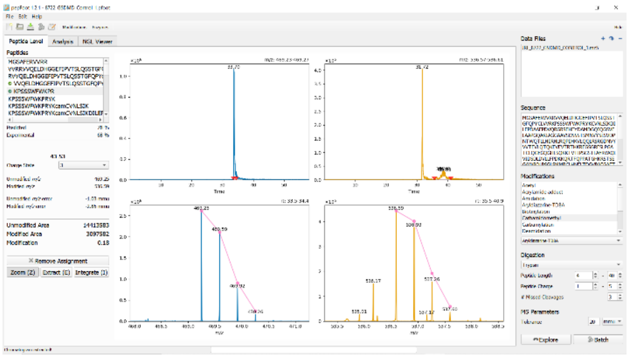
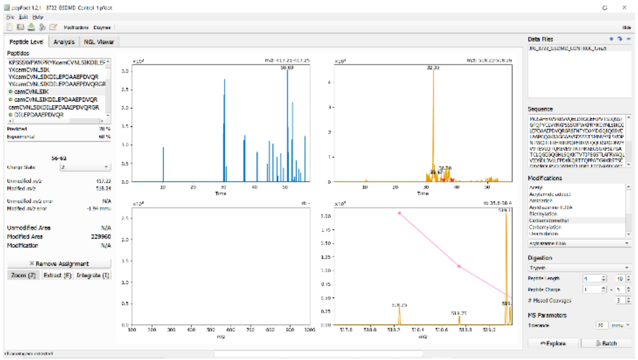
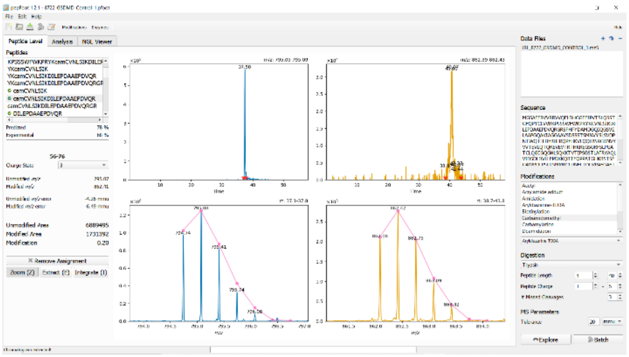
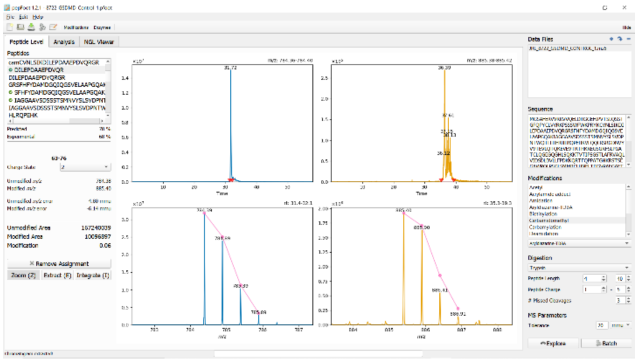
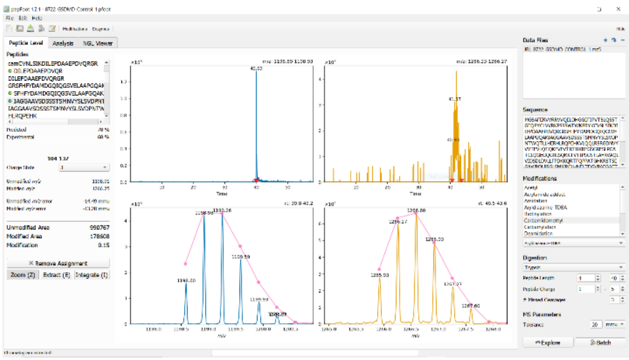
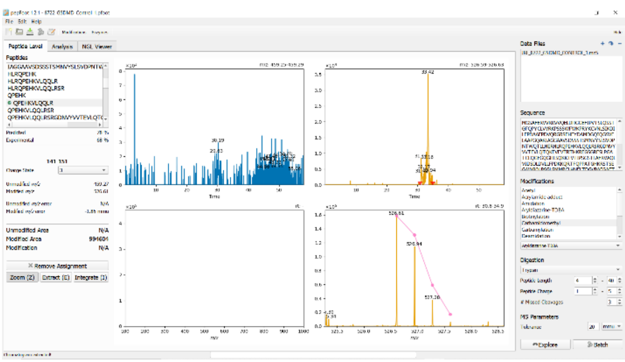
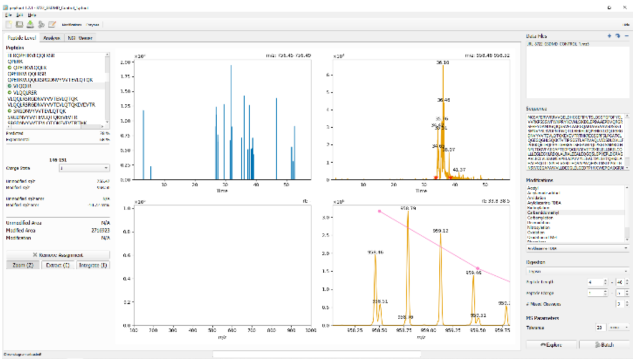

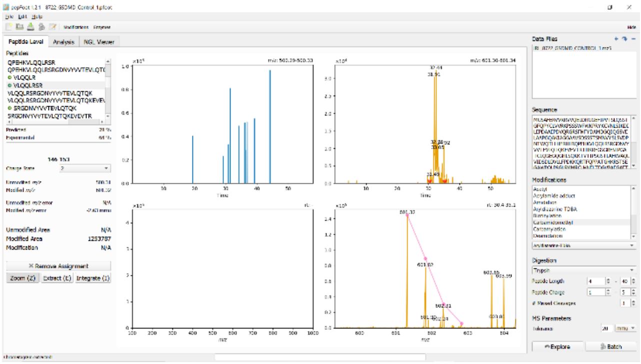
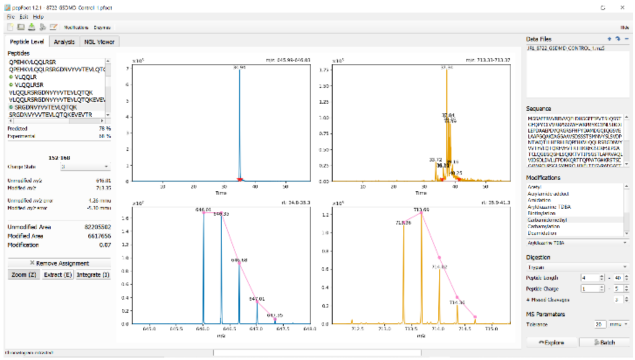
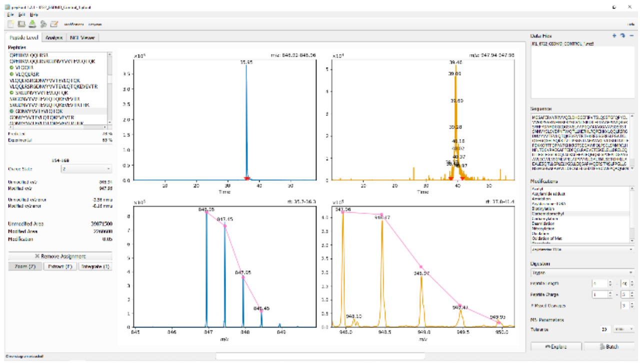
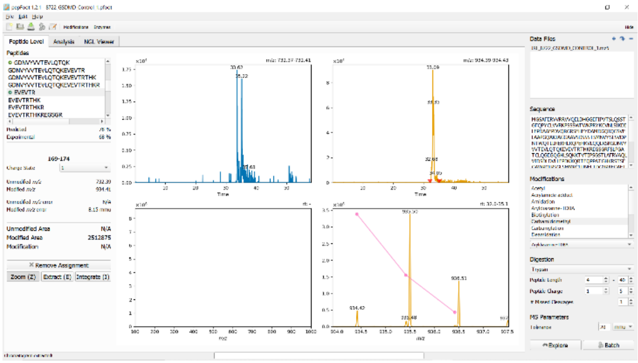
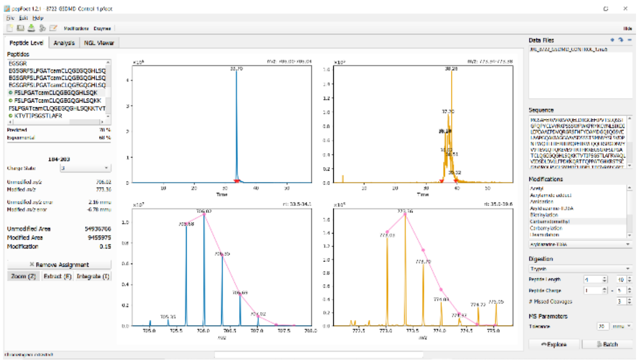
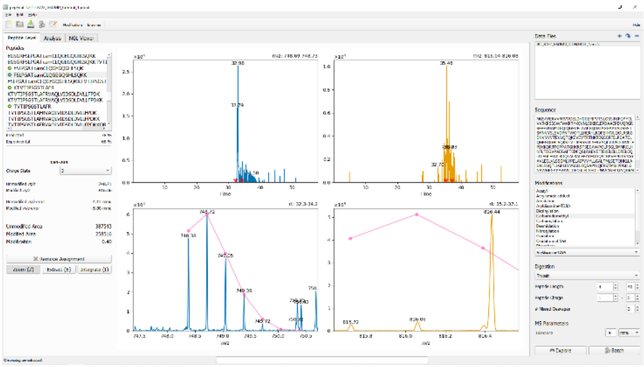
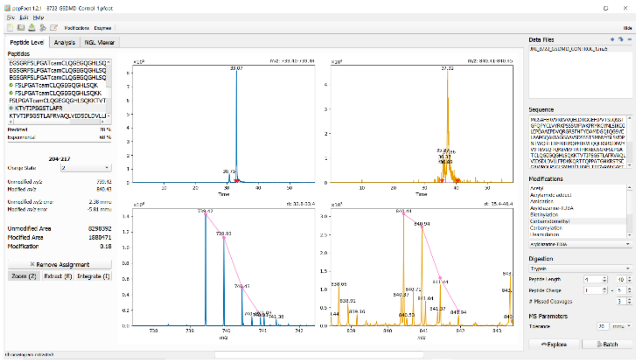
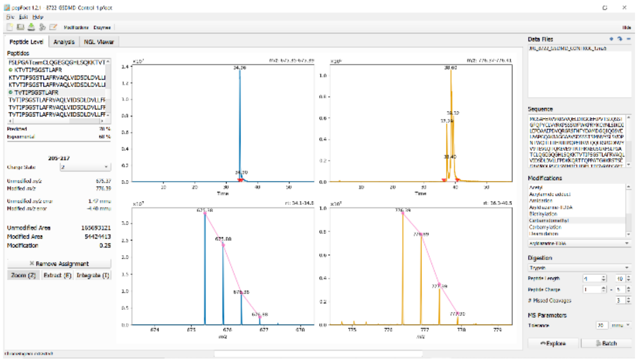
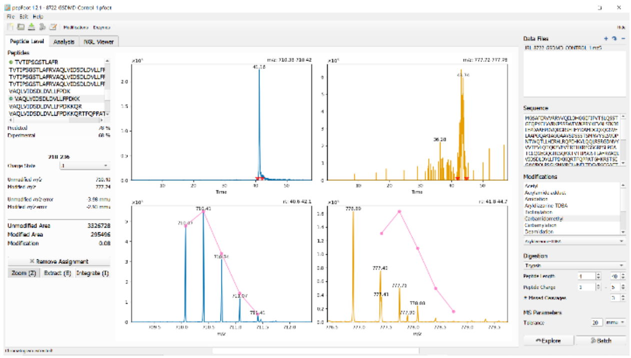
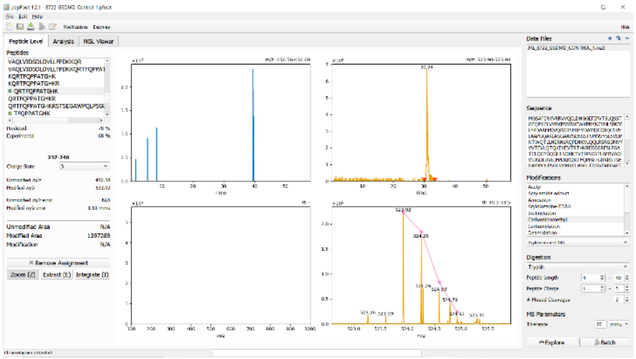

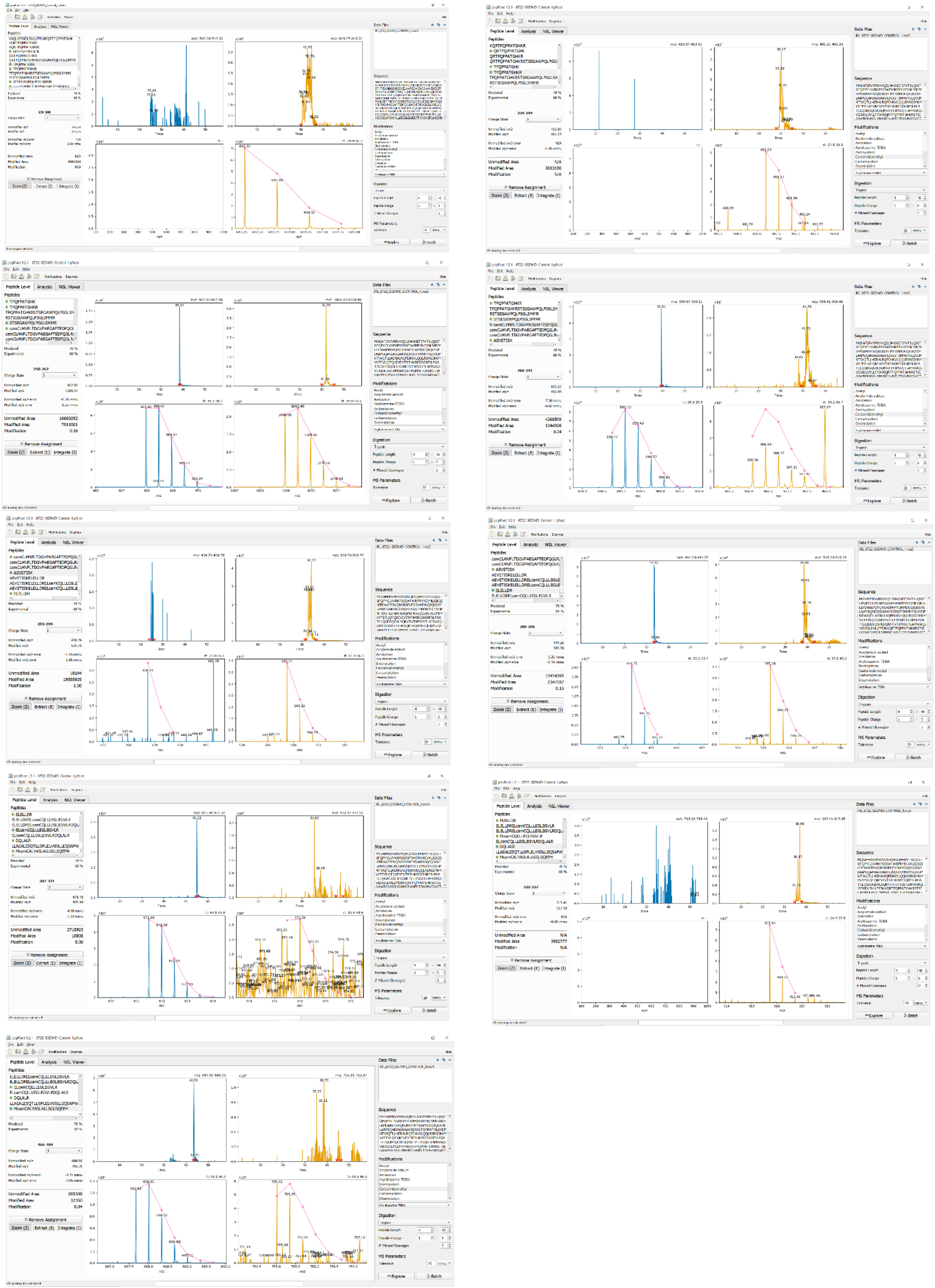

Figure S16. PepFoot extracted ion chromatogram assignments of GSDMD tryptic peptides from ‘control 1’ sample as an illustration.

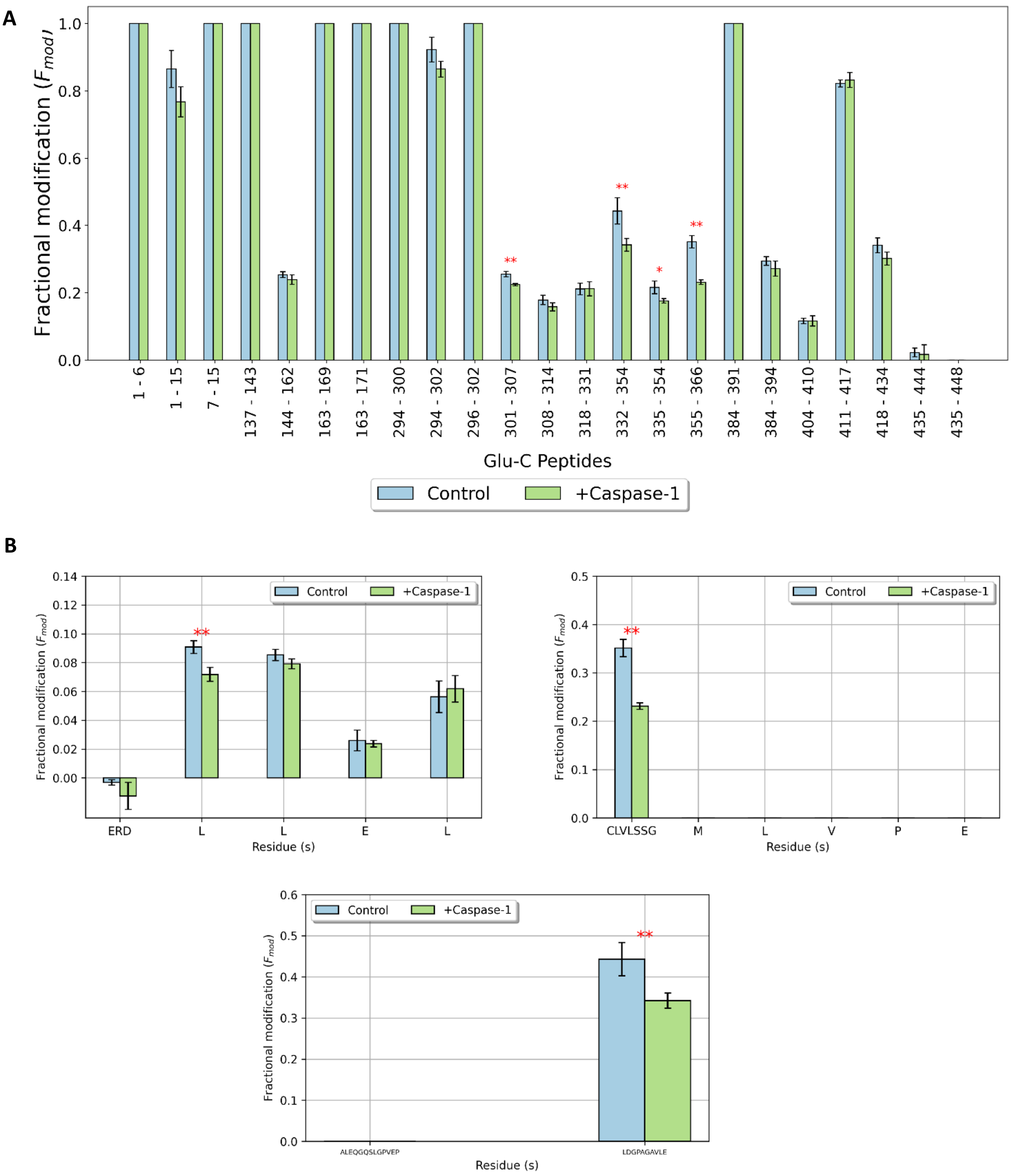

Figure S17. A) Fractional modification of Glu-C GSDMD peptides used in the differential study, with (green) and without (blue) 2:1 molar equivalents Caspase-1. Error bars are ± standard deviation (n = 4). Significant difference between samples is highlighted with ** (Student t-test, P < 0.01). B) Fractional modification of labeled peptides 301-307 (LELLDRE), 332-354 (ALEQGQSLGPVEPLDGPAGAVLE) and 355-366 (CLVLSSGMLVPE) following MS/MS fragmentation to reveal sub-peptide level localization. Error bars are ± standard deviation (n = 4). Significant difference between samples is highlighted with * (Student t-test, P < 0.05) or ** (Student t-test, P < 0.01).

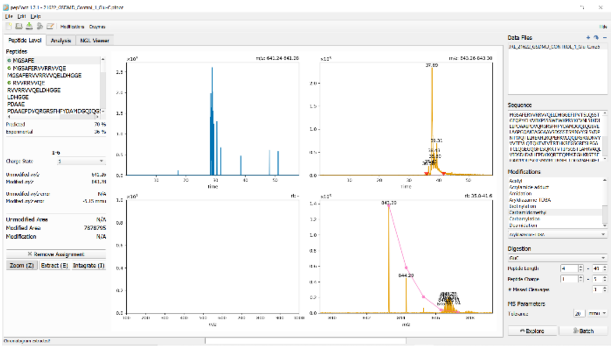
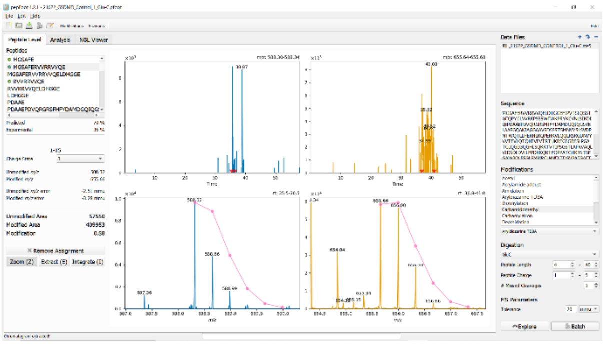
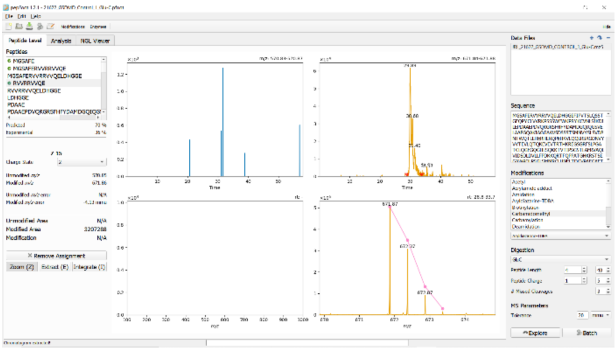
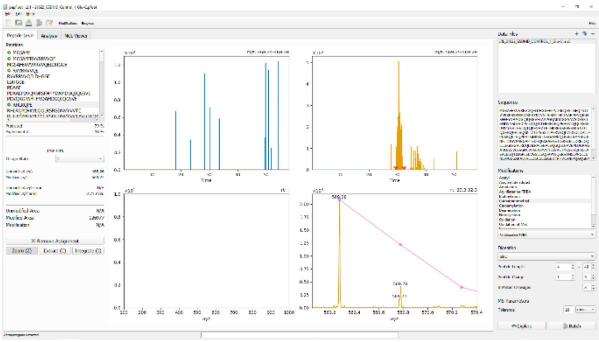
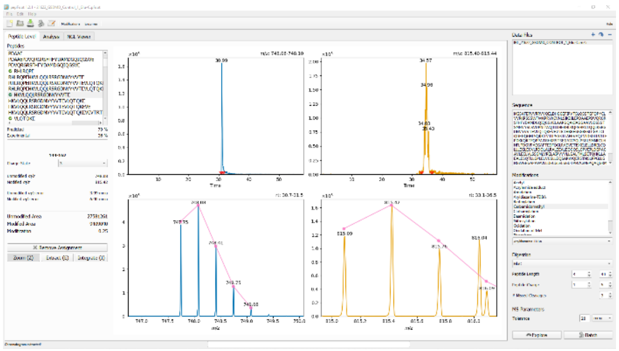
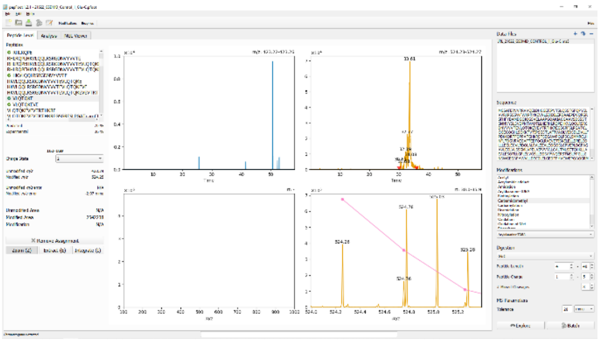
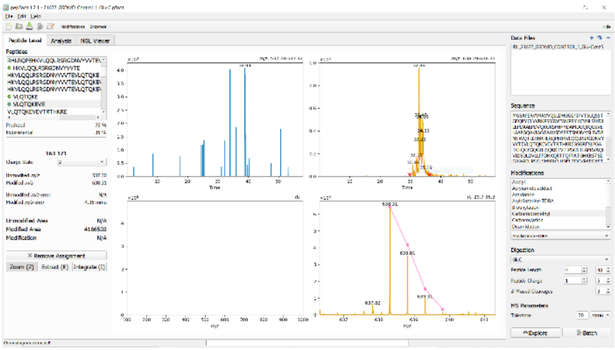
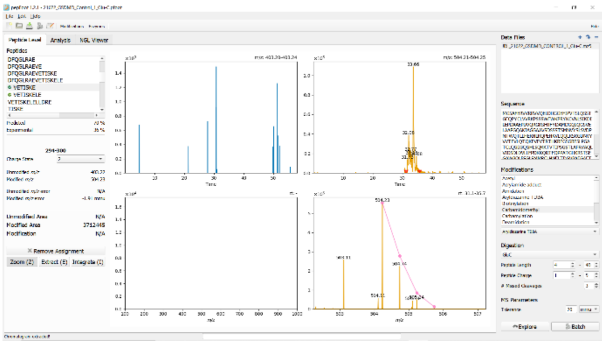
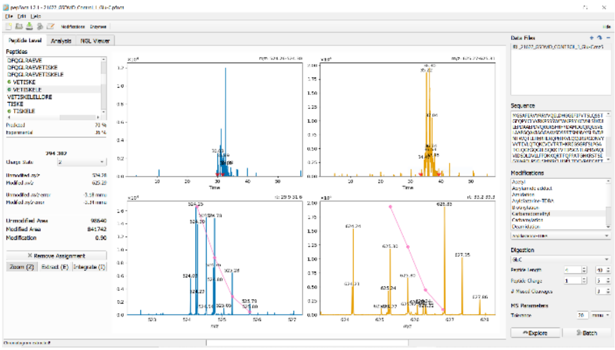
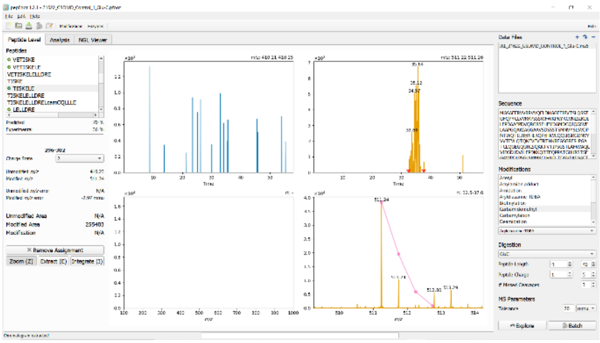

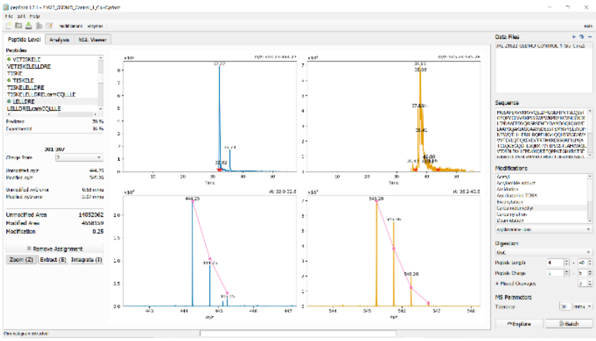
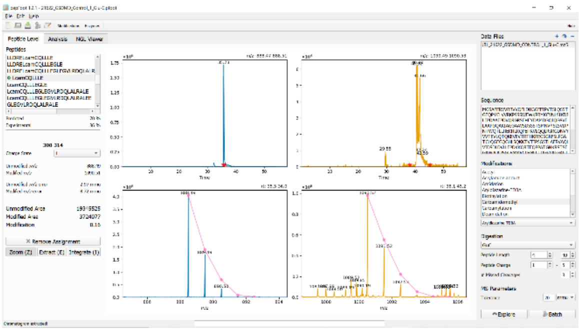
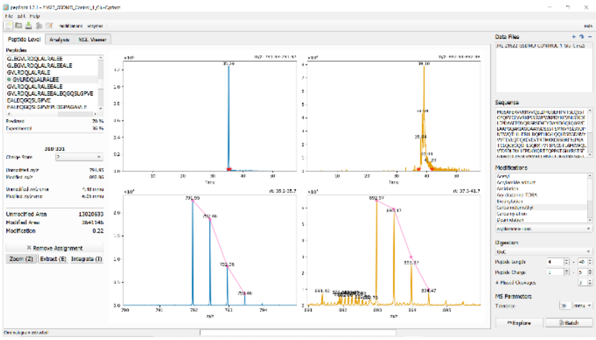
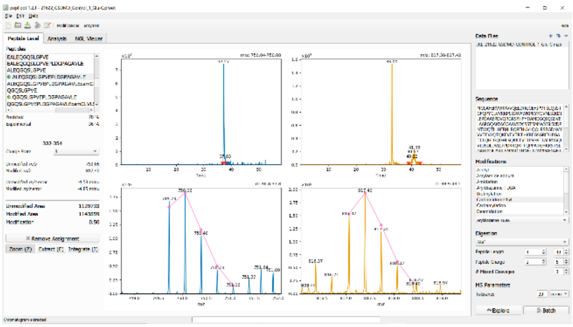
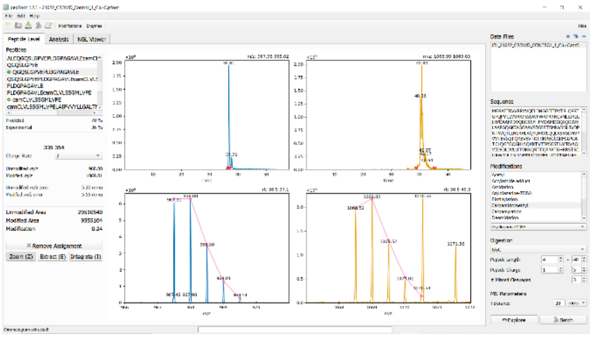
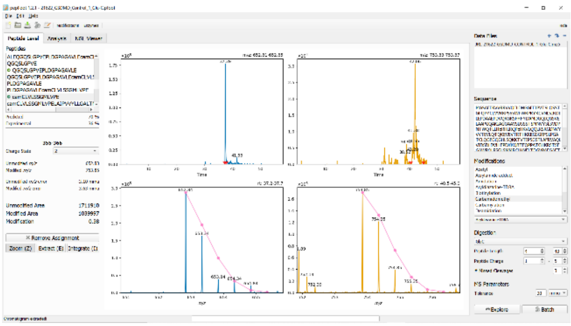
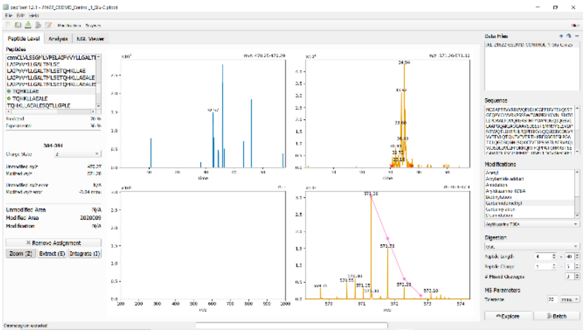
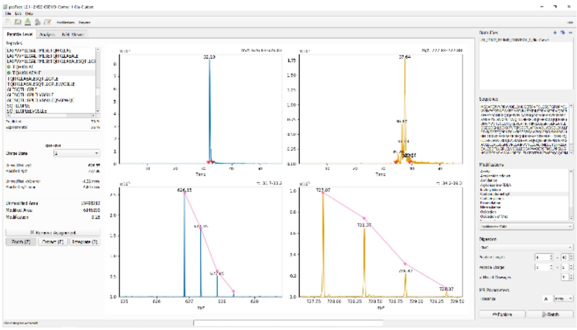
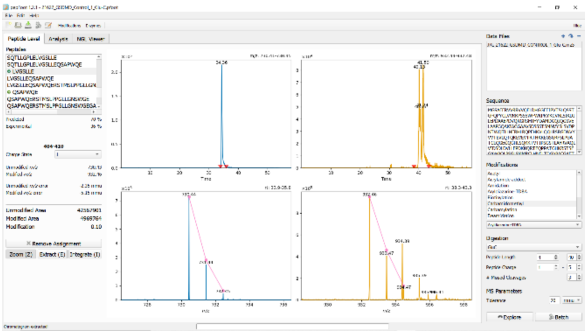
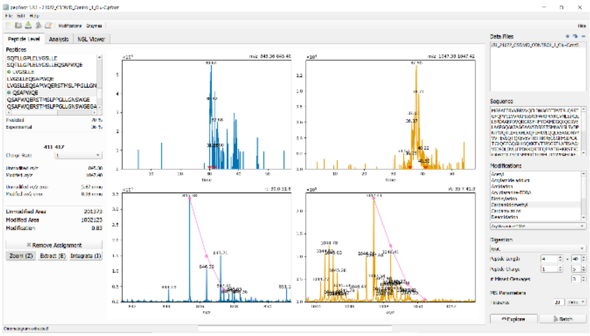

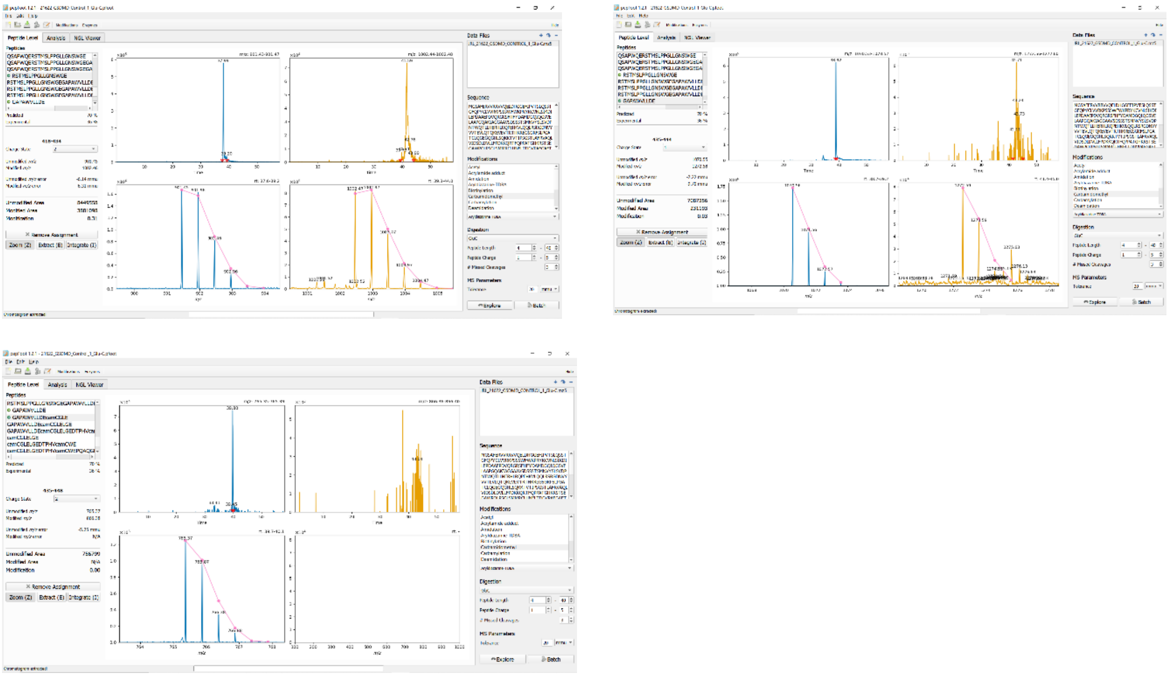

Figure S18. PepFoot extracted-ion chromatogram assignments of GSDMD Glu-C peptides from the ‘control 1’ sample for illustration.

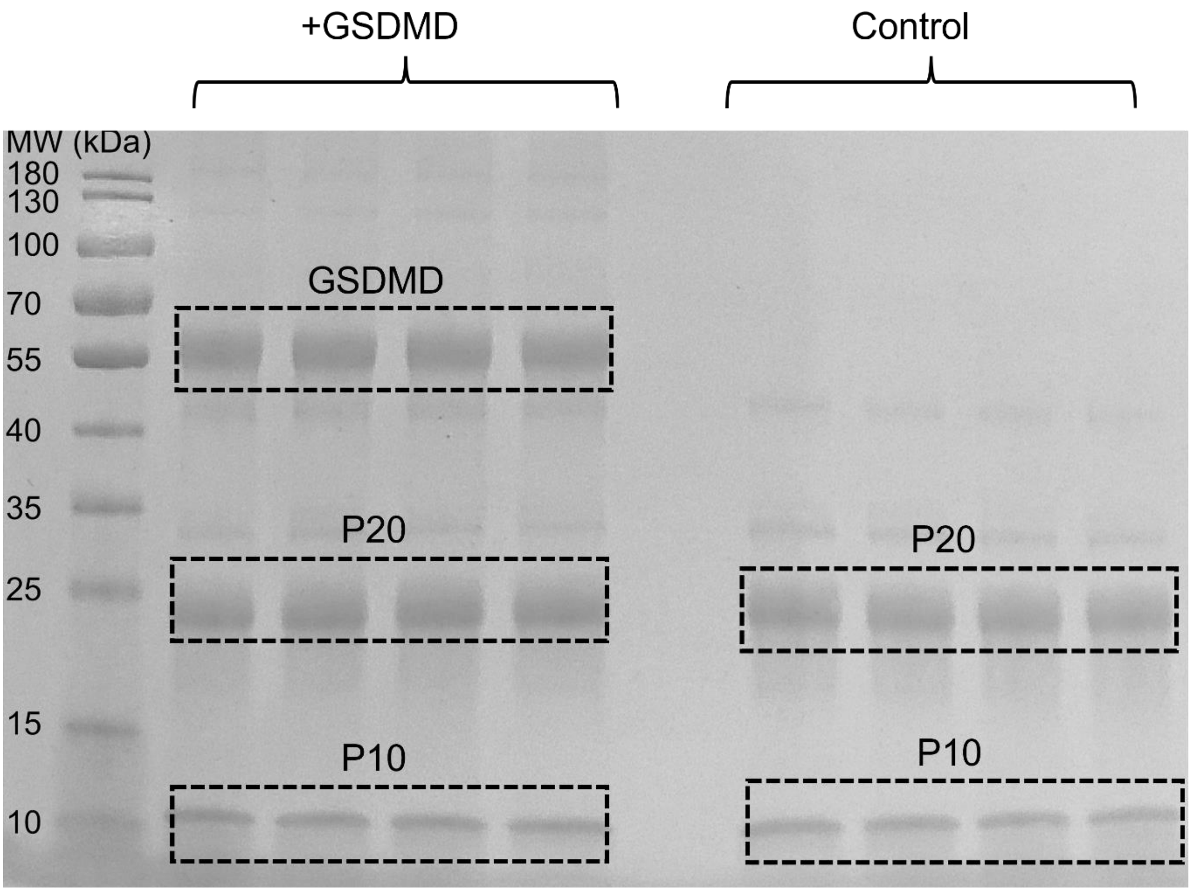

Figure S19. SDS-PAGE of labeled (20 mM NaTDB) Caspase-1 in the presence (left) and absence (right) of 2x molar equivalents of GSDMD (n = 4). Bands corresponding to the P10 and P20 subunits of Caspase-1 are labeled.

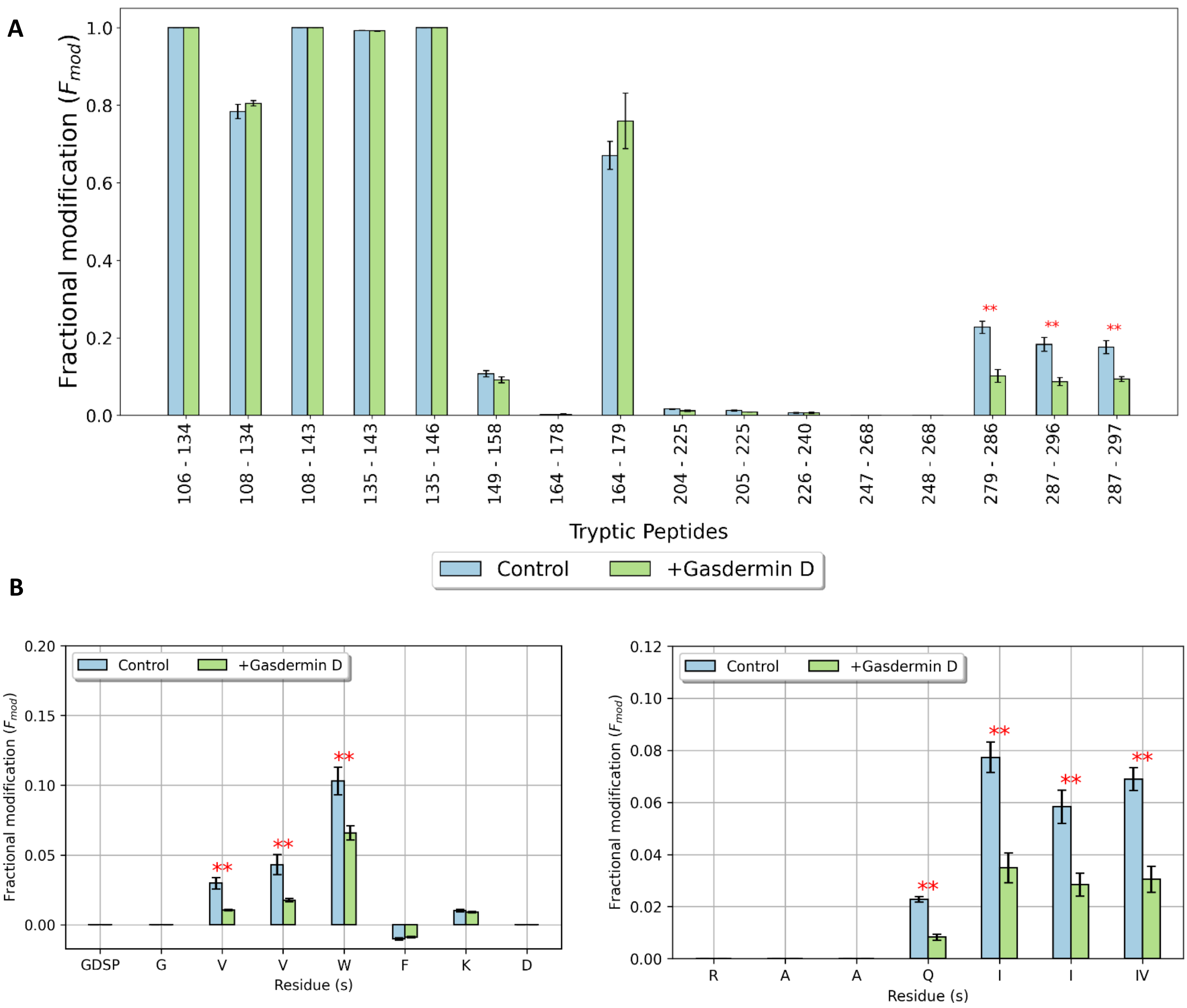

Figure S20. A) Fractional modification of tryptic Caspase-1 p20 subunit peptides used in the differential study, with (green) and without (blue) 2:1 molar equivalents GSDMD. Error bars are ± standard deviation (n = 4). Significant difference between samples is highlighted with ** (Student t-test, P < 0.01). B) Fractional modification of labeled peptide 279-286 (VIIIQAAR) and 287-297 (GDSPGVVWFKD) following MS/MS fragmentation to show sub-peptide level localization of the label. Error bars are ± standard deviation (n = 4). Significant difference between samples is highlighted with * (Student t-test, P < 0.05) or ** (Student t-test, P < 0.01).

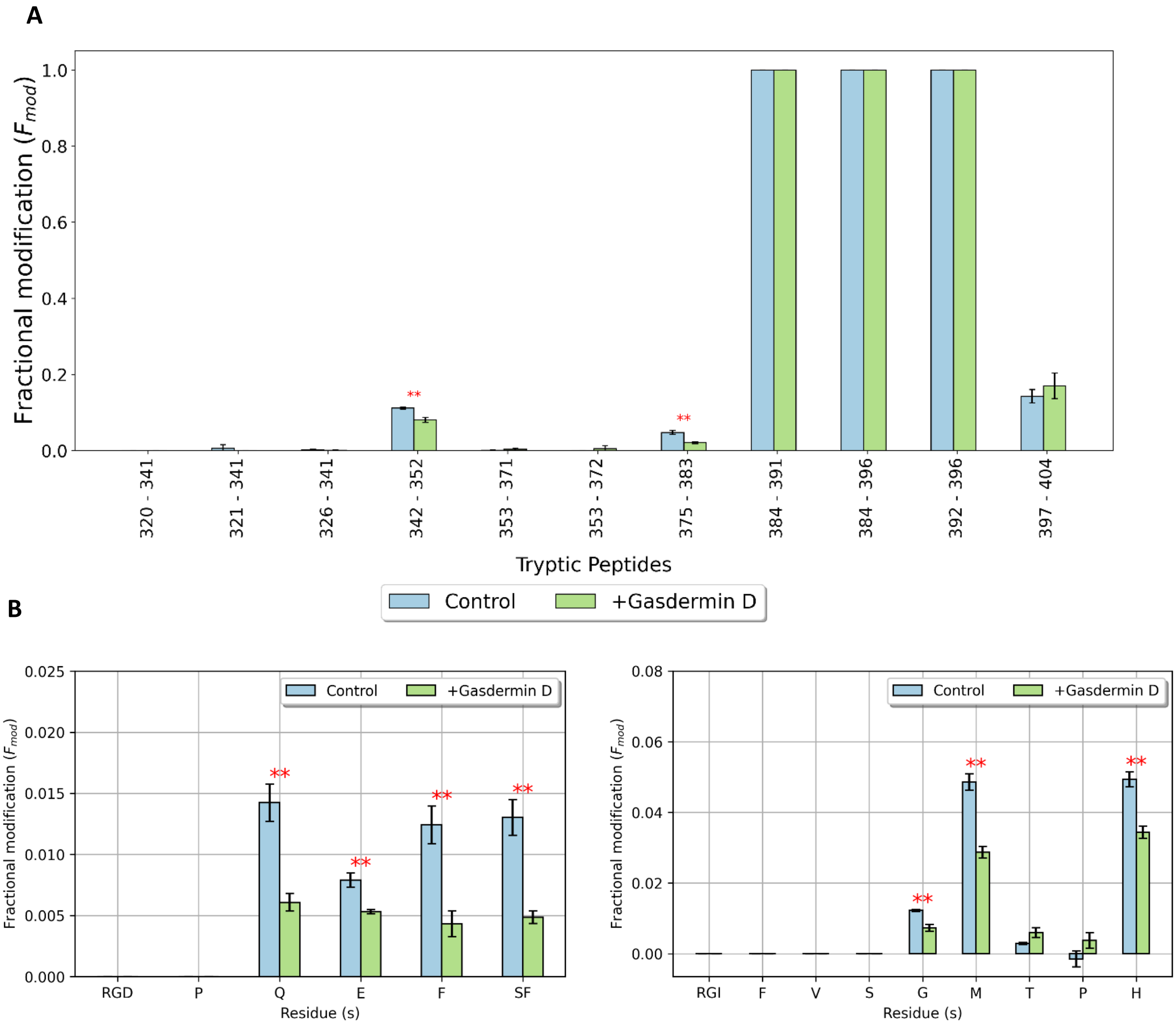

Figure S21. A) Fractional modification of tryptic Caspase-1 p10 subunit peptides used in the differential study, with (green) and without (blue) 2:1 molar equivalents GSDMD. Error bars are ± standard deviation (n = 4). Significant difference between samples is highlighted with ** (Student t-test, P < 0.01). B) Fractional modification of labeled peptides 342-352 (HPTMGSVFIGR) and 375-383 (FSFEQPDGR) following MS/MS fragmentation to show sub-peptide level localization of the label. Error bars are ± standard deviation (n = 4). Significant difference between samples is highlighted with * (Student t-test, P < 0.05) or ** (Student t-test, P < 0.01).

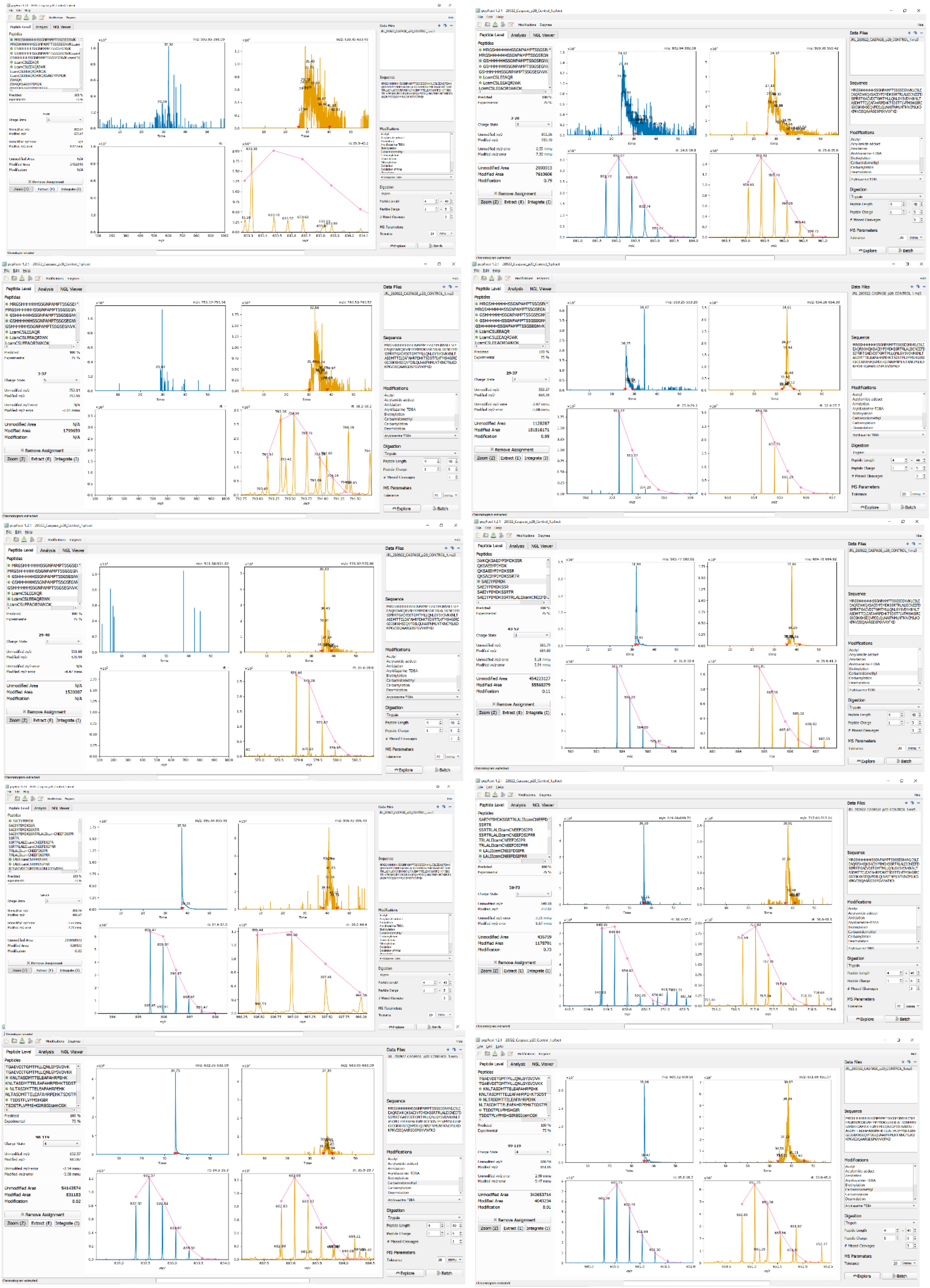

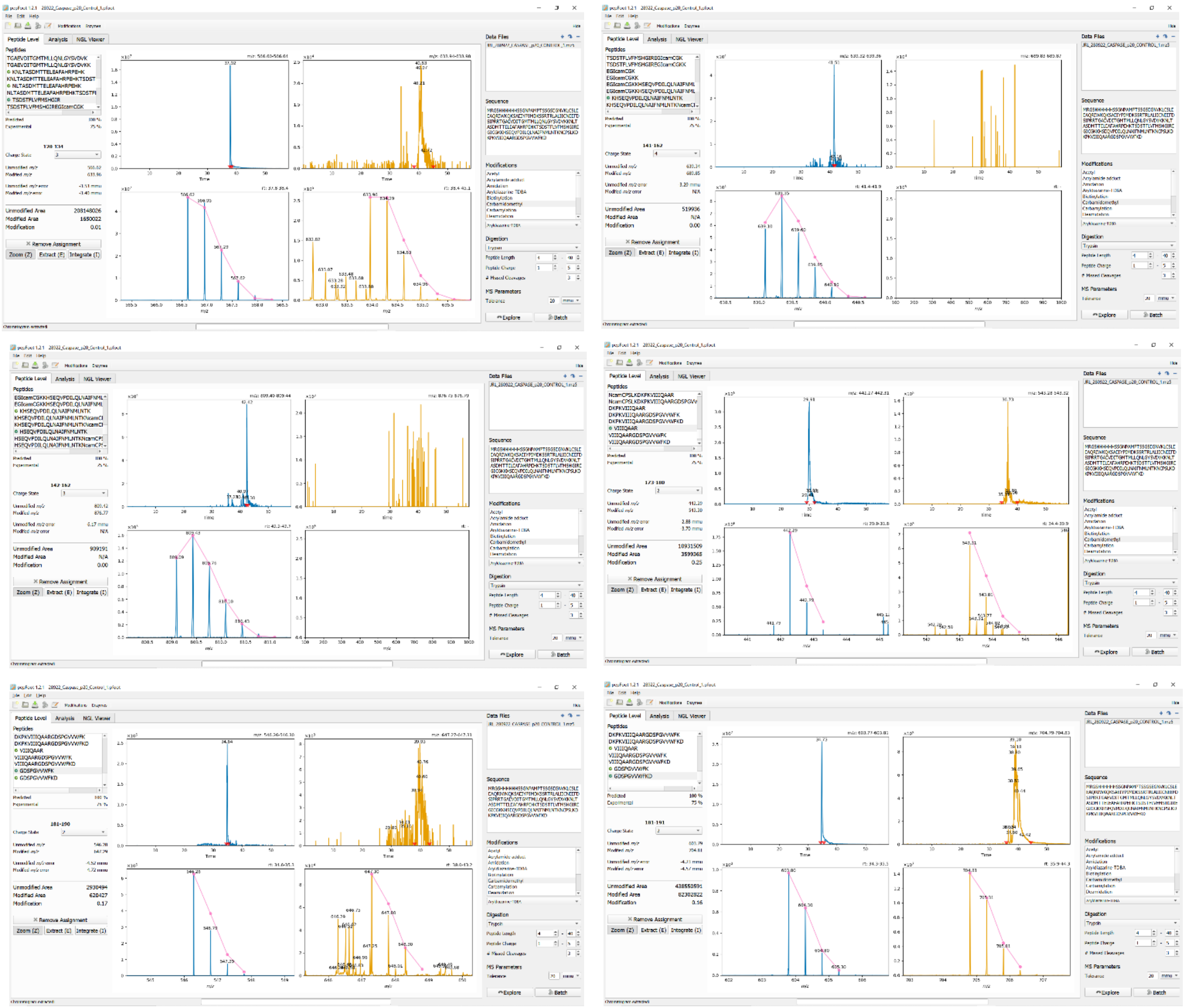

Figure S22. PepFoot extracted-ion chromatogram assignments of Caspase-1 p20 tryptic peptides from 'control 1' sample as an illustration.

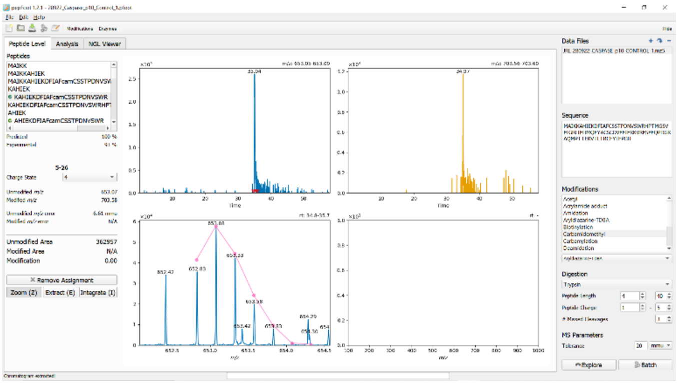

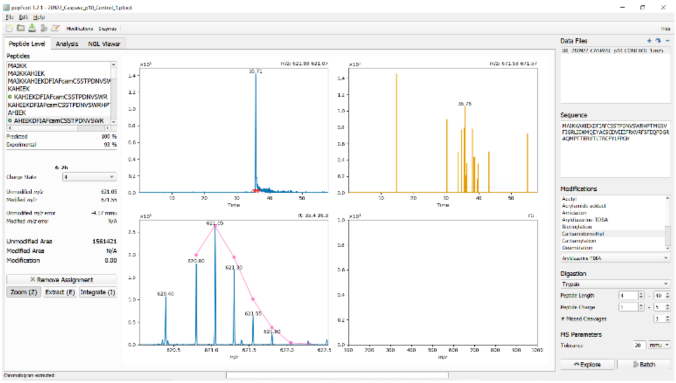

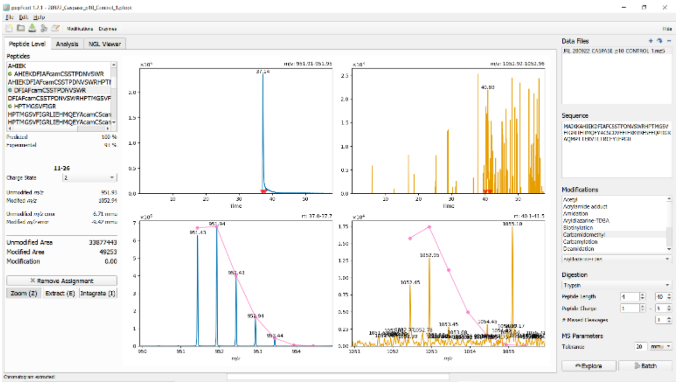

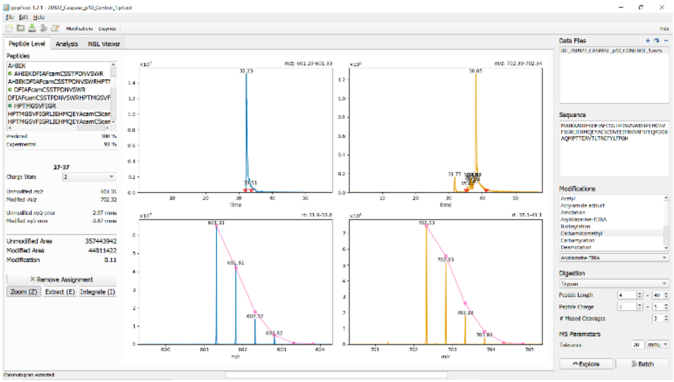

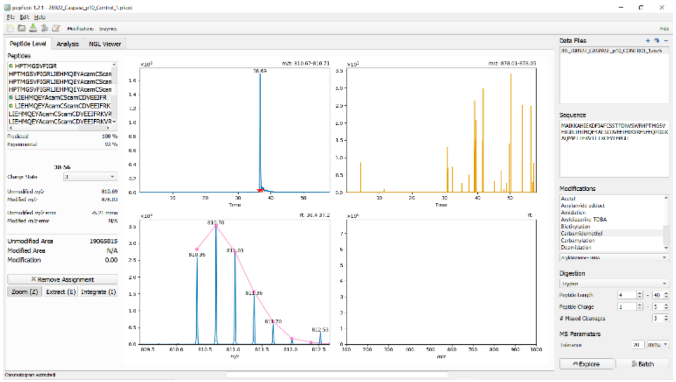

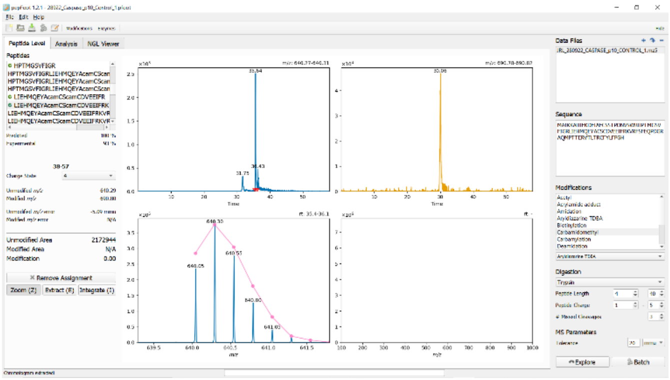

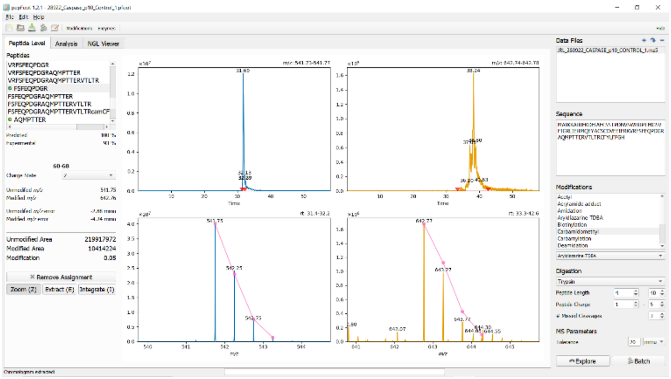

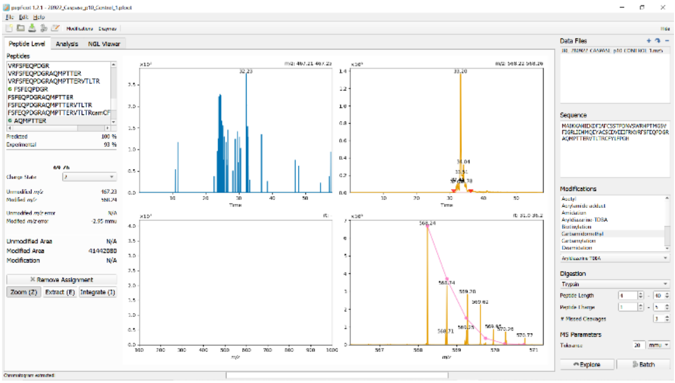

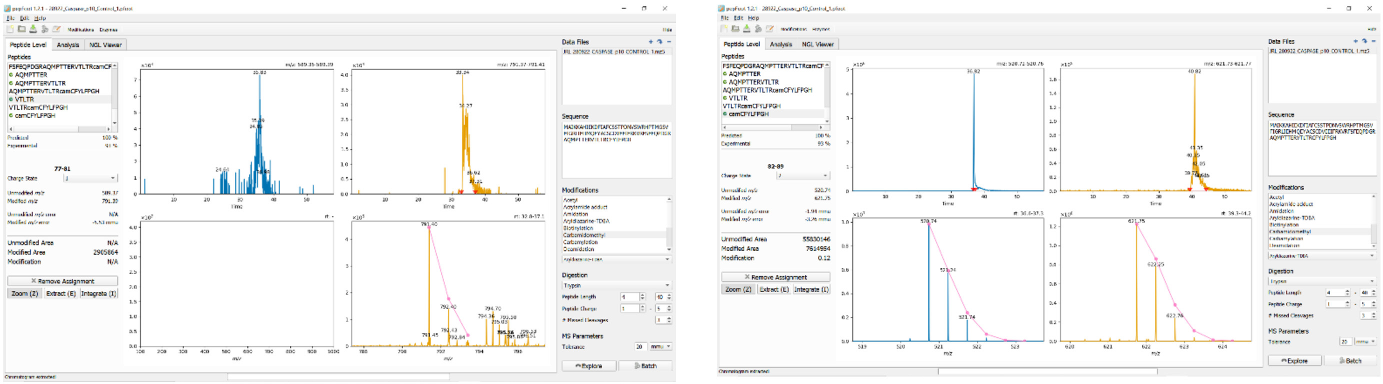

Figure S23. PepFoot extracted-ion chromatogram assignments of Caspase-1 p10 tryptic peptides from the ‘control 1’ sample for illustration.

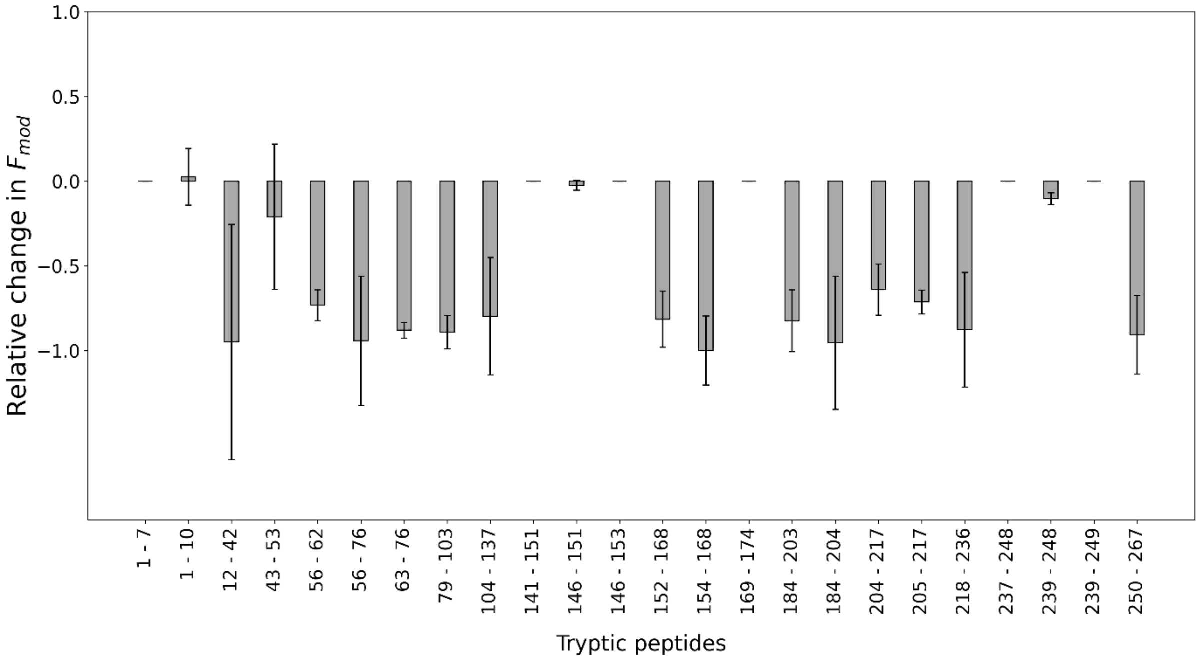

Figure S24. Relative change in fractional modification between labeled full-length GSDMD and GSDMD-NT pores.

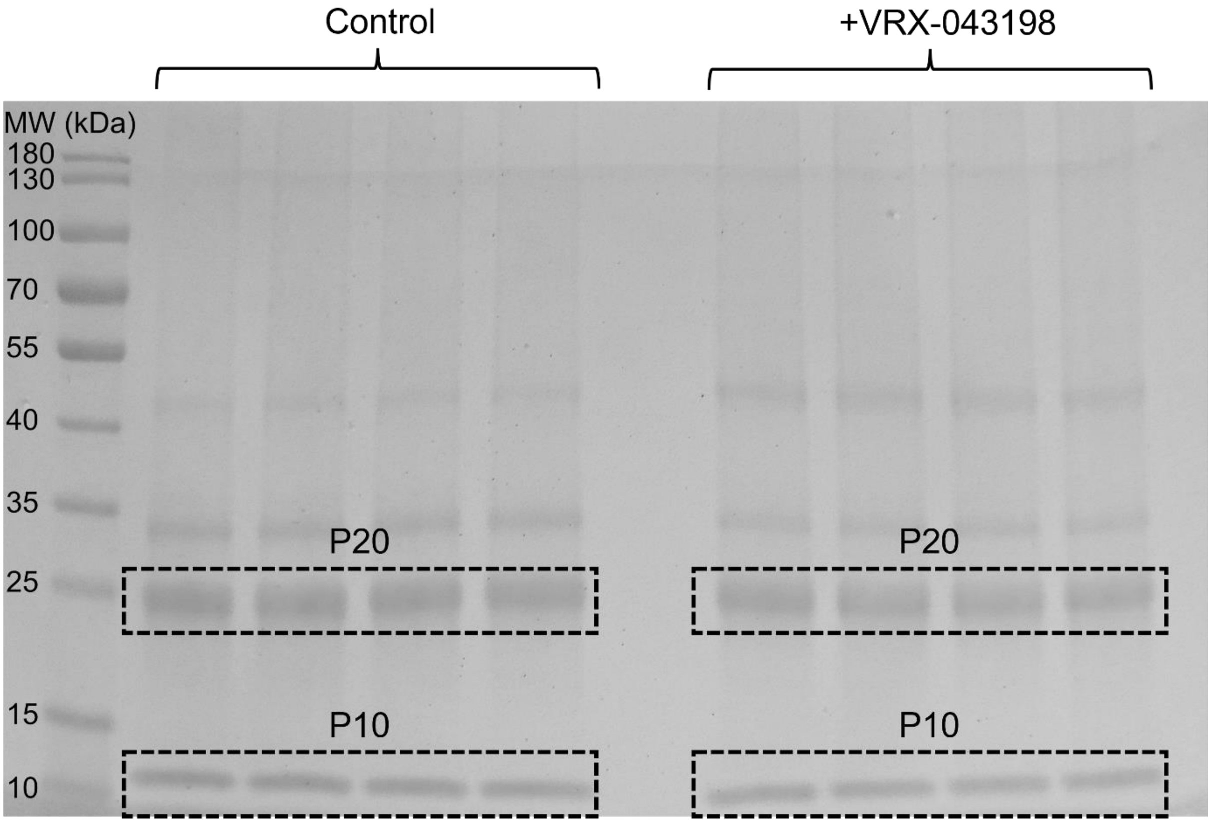

Figure S25. SDS-PAGE of labeled (20 mM NaTDB) Caspase-1 in the presence (right) and absence (left) of 100 uM VRT-043198 (n = 4). Bands corresponding to Caspase-1 P10 and P20 subunits are labeled.

A

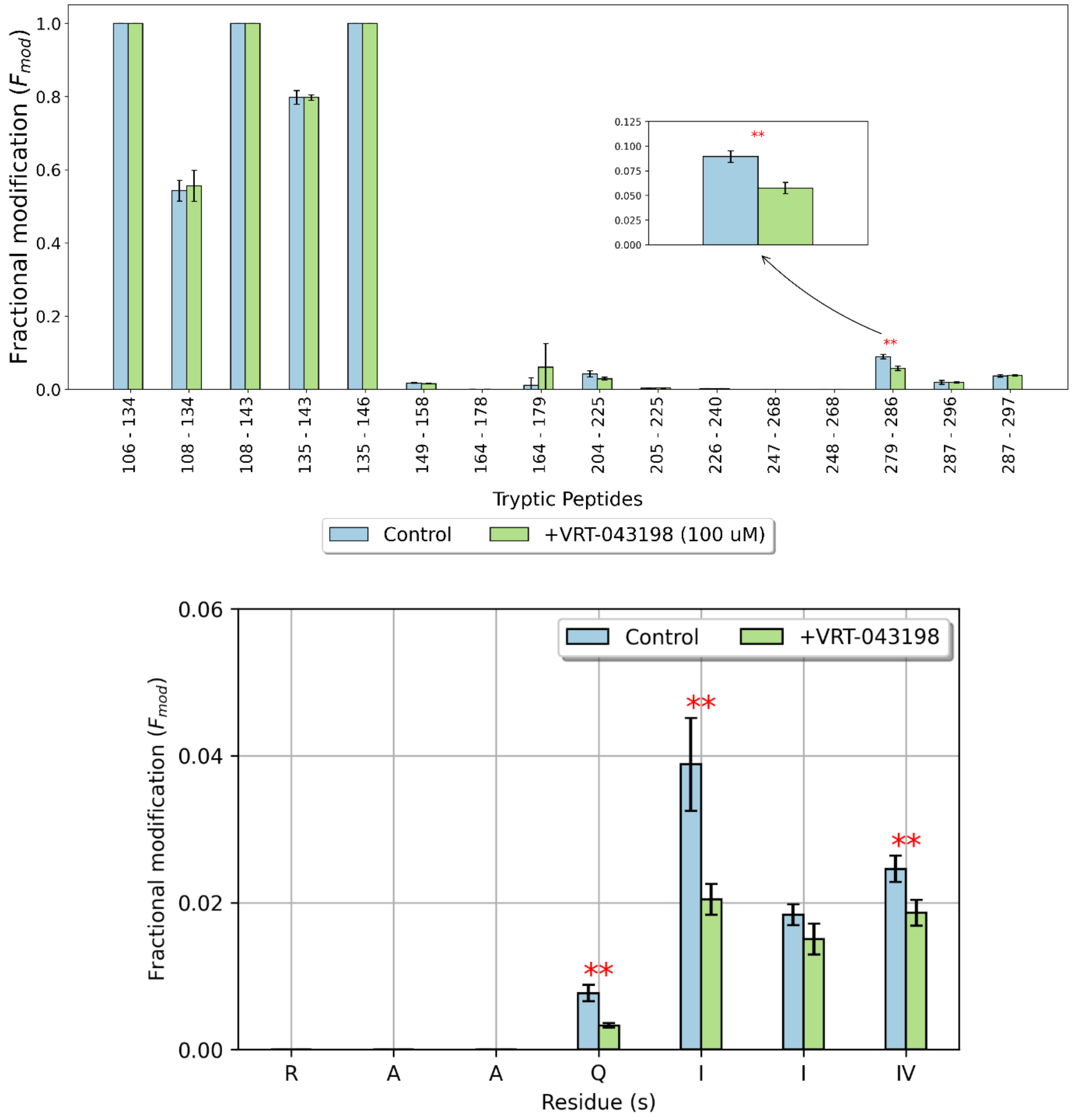

Figure S26. A) Fractional modification of tryptic Caspase-1 p20 subunit peptides used in the differential study, with (green) and without (blue) 100 µM VRT-043198. Error bars are ± standard deviation (n = 4). Significant difference between samples is highlighted with ** (Student t-test, P < 0.01). B) Fractional modification of labeled peptide 279-286 (VIIIQAAR) following MS/MS fragmentation to show sub-peptide level localization of the label. Error bars are ± standard deviation (n = 4). Significant difference between samples is highlighted with * (Student t-test, P < 0.05) or ** (Student t-test, P < 0.01).

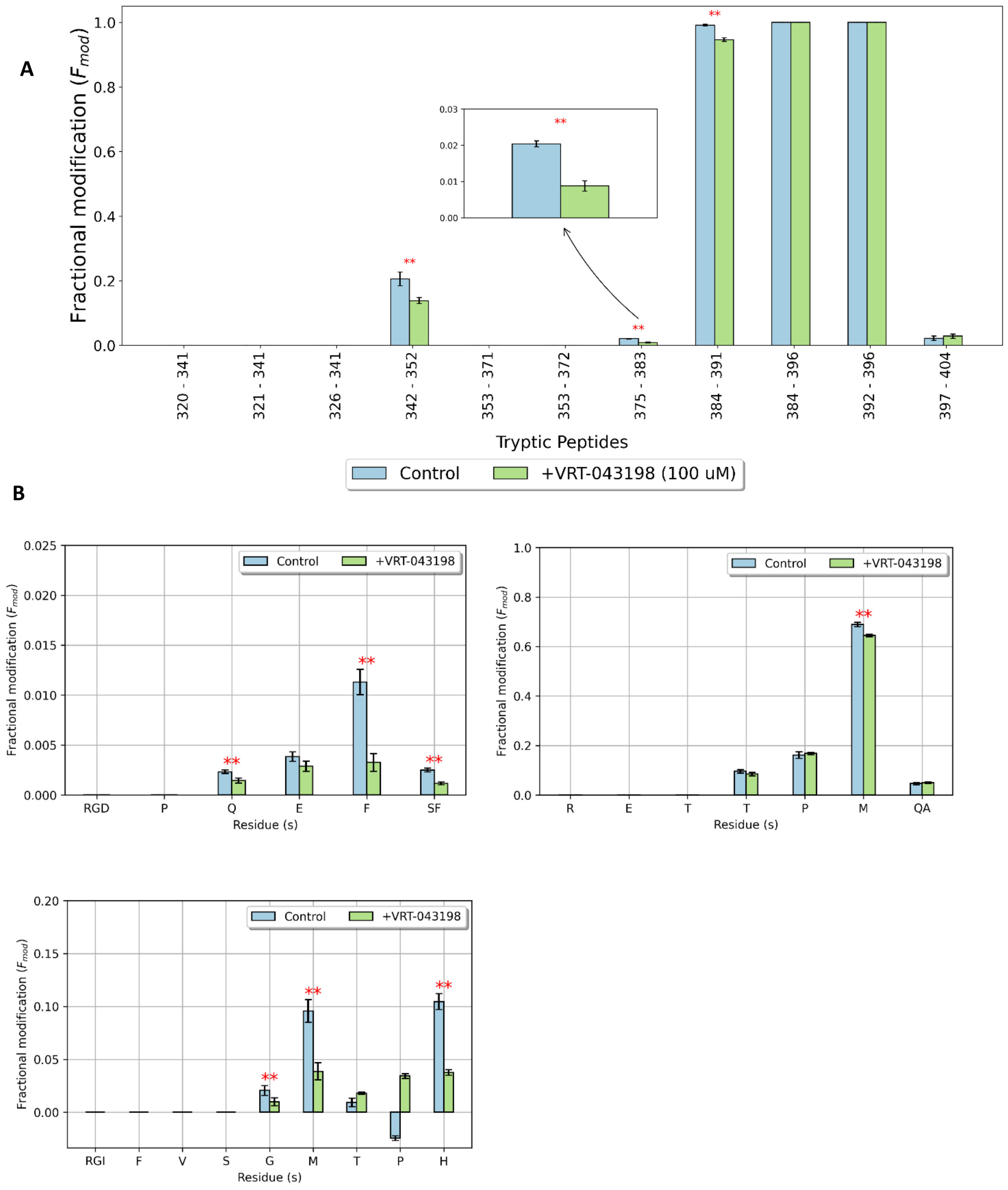

Figure S27. A) Fractional modification of tryptic Caspase-1 p10 subunit peptides used in the differential study, with (green) and without (blue) 100 µM VRT-043198. Error bars are ± standard deviation (n = 4). Significant difference between samples is highlighted with ** (Student t-test, P < 0.01). B) Fractional modification of labelled peptide 342-352 (HPTMGSVFIGR), 375-383 (FSFEQPDGR) and 384-391 (AQMPTTER) following MS/MS fragmentation to show sub-peptide level localization of the label. Error bars are ± standard deviation (n = 4). Significant difference between samples is highlighted with * (Student t-test, P < 0.05) or ** (Student t-test, P < 0.01).

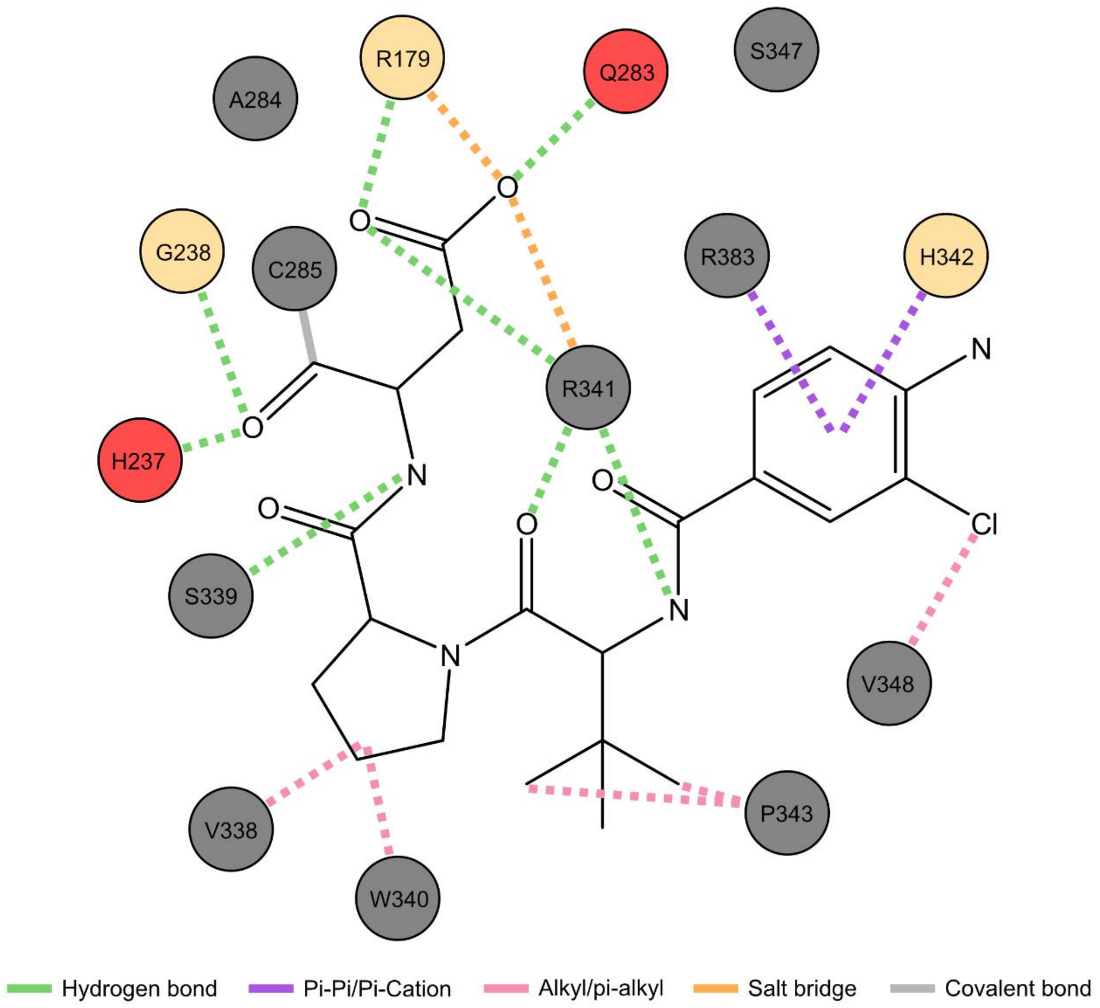

Figure S28. Predicted binding contacts between Caspase-1 and VRT-043198. Residue coloring according to labelling data. Adapted from Li and colleagues (29).
